# Plastic additives enrich diverse bacterial communities which show the hallmarks of plastic degradation

**DOI:** 10.1093/sumbio/qvag003

**Published:** 2026-01-28

**Authors:** Matthew J Tarnowski, Andy Stawowy, Eva C Sonnenschein

**Affiliations:** Department of Biosciences, Swansea University, Singleton Park, Swansea SA2 8PP, United Kingdom; Department of Biosciences, Swansea University, Singleton Park, Swansea SA2 8PP, United Kingdom; Department of Biosciences, Swansea University, Singleton Park, Swansea SA2 8PP, United Kingdom

**Keywords:** microbiome, amplicon sequencing, microbial biotechnology, high-throughput, enrichment culture, plastic degradation

## Abstract

Plastics contain a variety of chemical additives that enhance their performance but often pose environmental risks due to their persistence and leaching. Microbial degradation offers a promising strategy to mitigate these pollutants, yet efficient methods to identify active degraders remain limited. This project aims to combine biochemical assays with 16S rRNA amplicon sequencing to screen microbial communities for plastic additive biodegradation. Inocula from natural (Chessel Bay, Swansea Bay) and anthropogenic (Wastewater Plant, Recycling Plant) environments were enriched for 22 days, using di(2-ethylhexyl) terephthalate (DEHT) and tetradecane and compared to negative controls. We adapted high-throughput assays to measure community-level growth, death, redox, and esterase activity. DEHT yielded the highest growth, while tetradecane enhanced redox activity. 16S rRNA amplicon sequence analysis identified 957 amplicon sequence variants across 36 cultures. PERMANOVA showed that the substrate explained 39%–63% of the variance in community structure. Both additives enriched bacterial families known to degrade plastics (e.g. *Nocardiaceae*, which correlated with esterase activity). Other bacterial families not previously associated with plastic degradation (e.g. *Vermiphiliaceae*) highlight potential for plastic and additive biodegradation. These results demonstrate that diverse environmental microbiomes can metabolize ester- and alkane-based plastic additives. Our methods enable scalable screening of biodegradative communities for bioremediation applications.

**Sustainability Statement**

This study supports progress toward UN Sustainable Development Goal 6 (Clean Water and Sanitation) by identifying microbial communities capable of degrading plastic additives—emerging pollutants that leach into aquatic systems. By developing a scalable, low-cost platform for profiling microbial biodegradation of ester- and alkane-based additives, we enable screening and prioritization of microbial communities for applications in wastewater treatment. These findings are already being applied within the BMRex project to inform the development of novel wastewater technologies that reduce microplastic loads.

## Introduction

Plastics are complex mixtures of chemicals containing many additives that modify the properties of the main polymer. These additives are only embedded in the polymer and therefore can leach into the environment over time (Rani et al. 2015, Xu et al. 2024, 2025). At least 27 Mt of plastic additives are used globally per year in plastic production (Wiesinger et al. 2021, Williams and Rangel-Buitrago 2022). Over 10 000 chemicals are used as plastic additives and many of these substances are poorly regulated and potentially hazardous (Wiesinger et al. 2021). Phthalate acid esters (PAEs) are a common additive in PVC and PET, with an estimated annual production of 8 Mt, and are often incorporated in plastic at 10%–60% by weight to enhance flexibility and durability (Khoshmanesh et al. 2024). Exposure to PAEs can be associated with infertility in men and women (Caporossi et al. 2020, Zhan et al. 2022).

Currently, the most common PAE in the environment is di(2-ethylhexyl) phthalate (DEHP) (Wowkonowicz 2023) and 3–4 Mt of this plastic additive are produced annually (Chen et al. 2023). Although DEHP can be synthesized naturally by red algae (Chen 2004), ambient levels are negligible compared to anthropogenic sources. In a meta-analysis of 59 articles studying phthalates in dust, DEHP was routinely the most abundant phthalate and on average it accounted for 0.06% of dust by mass (Bu et al. 2020). The toxic effects of DEHP on the endocrine, testicular, ovarian, neural, hepatic and cardiac systems are comprehensively reviewed elsewhere (Rowdhwal and Chen 2018). DEHP is amongst many additives released into the environment, for example, from mulching films in soil (Xu et al. 2024) and its half-life in soil is estimated to be 50–300 days (Kim et al. 2019) demonstrating its degradation. In the environment, DEHP levels range from nanomolar in surface waters, to low micromolar in soil, wastewater and landfill leachates, to low millimolar in sewage sludge (Wirnitzer et al. 2011).

DEHT (a *para-*phthalate) is a structural isomer of DEHP (an *ortho-*phthalate), which is increasingly being used in plastic production as an alternative to DEHP, as evidenced by its rising presence in dust samples (Qadeer et al. 2024). However, DEHT has been highlighted as potentially toxic to aquatic organisms (Qadeer et al. 2024) and showed carcinogenicity and chronic toxicity following a 2-year dietary exposure in rats, which found the no-observed effect level for tumorigenicity and chronic toxicity to be 12 g/kg and 1.5 g/kg, respectively (Deyo 2008).

In controlled laboratory settings, microbial isolates such as *Enterobacter* species YC-IL1 (Lamraoui et al. 2020), *Mycobacterium* sp. NK0301 (Bhattacharyya et al. 2023), *Acinetobacter* sp. SN13 (Xu et al. 2017), *Ochrobactrum anthropi* L1-W (Nshimiyimana et al. 2020), *Gordonia alkanivorans* YC-RL2 (Nahurira et al. 2017), *Gordonia polyisoprenivorans* G1, *Rhodococcus rhodochrous* G7, and *Corynebacterium nitrilophilus* G11 (Chao and Cheng 2007) are capable of effectively removing DEHT from aqueous solutions within weeks. However, much less is known about how entire microbial communities respond to DEHT. Saeng-kla et al. found that mangrove sediment microbiomes affected by chronic plastic pollution degraded 99% of 200 mg/kg DEHT after 30 days in sediment microcosms (Saeng-kla et al. 2025). Analysis of the microbial diversity using 16S rRNA amplicon sequencing suggested that *Myxococcales, Methyloligellaceae, Mycobacterium*, and *Micromonospora* were potentially responsible for DEHT degradation. A further study identified a bacterial consortium including *Paraclostridium, Staphylococcus*, and *Bacillus* that grew in the presence of environmentally relevant concentrations of DEHT (6 mg/L) (de Morais Farias et al. 2025). Understanding the capacity of environmental microbiomes to adapt to plastic additives, such as DEHT, is crucial in the context of both plastic additive release in the environment and plastic recycling, which releases high concentrations of plastic breakdown products and additives (Schaerer et al. 2024).

Enrichment cultures are a valuable tool for studying microbial communities capable of degrading target pollutants. This approach overcomes the limitations of shotgun environmental metagenomics, which often infers rather than demonstrates function, and of pure culture studies, which neglect synergistic interactions essential for degradation. 16S rRNA gene amplicon analysis can then provide insights into microbial community composition (Caporaso et al. 2010), however, it does not directly assess metabolic activity (Douglas et al. 2020). While shotgun metagenomics, metatranscriptomics, and metaproteomics offer direct insights into gene expression during plastic biodegradation, they can be prohibitively expensive. Therefore, utilization of low-cost functional biochemical assays, such as those measuring cell growth, enzyme activity, and metabolite production, is essential for linking microbial community structure to function and permits study of multiple environmental samples in parallel.

Increased microbial growth in the presence of a plastic substrate can indicate metabolism of the substrate. Using cell-permeable and cell-impermeable nucleic acid dyes, total and dead cell biomass can be estimated (Martens-Habbena and Sass 2006). Though this is not definitive evidence for biodegradation, cell growth, and death can indicate active metabolism or toxicity, respectively. Many plastic additives have the same chemical functional groups (e.g. alkane and ester) as plastic polymers, enabling the repurposing of assays suited to monitoring plastic degradation. Furthermore, this may mean that plastic additive-degrading species have increased potential for plastic degradation. For polyolefins, assays have been developed to monitor the multiple steps of the biodegradation pathway, which begins with oxidation via formation of extracellular reactive oxygen species (ROS) (Zadjelovic et al. 2022). Redox-active dyes can indicate the occurrence of this first step in plastic biodegradation (Kim et al. 2023). Meanwhile for polyesters, esterase activity is most relevant and can be measured using ester-containing dyes which change color upon hydrolysis of the ester bond (Liu et al. 2023). These simple dye-based biochemical assays can be undertaken in a high-throughput microplate format that allows 100 s of measurements to be taken simultaneously using a fluorescent plate-reader, allowing many samples to be assessed simultaneously (Yew et al. 2025). Finally, while lower in throughput than biochemical assays, liquid chromatography–mass spectrometry (LC–MS) can detect extremely low concentrations of plastic breakdown products, enabling confirmation of biodegradation. However, LC–MS has several limitations such as contamination and metabolite-specific sample preparation, which means that establishing and running a protocol is time-intensive and costly. While high-throughput screening can enable rapid assessment of various combinations of plastic additive and inoculant, LC–MS remains valuable as a tool to triage screening hits and confirm their activity.

This study aims to establish high-throughput pipeline to prioritize microbial communities for biodegradation of plastic additives. Two environmental inocula from anthropogenic environments, with high ambient plastic levels (Wastewater Plant and Recycling Plant) and two from natural environments (Swansea Bay, Chessel Bay) with intermittent plastic contamination were enriched on the plastic additives DEHT and tetradecane and compared to a control. The half-life for the biodegradation of phthalates and long-chain alkanes by environmental inocula is in the region of days to years (Prince et al. 2017, Saeng-kla et al. 2025) and increases with concentration. We incubated the enrichment cultures for 22 days at 30°C with each substrate at a concentration over 10-fold higher than environmentally-relevant levels, to maximize the chance of identifying microbial consortia capable of biodegradation.

Tetradecane is a long chain alkane, which can be used as raw material for plastic production, solvent during polymerization and additive to alter plastic properties. The two additives used in this study—DEHT and tetradecane—contain chemical structures representative of major plastic classes. DEHT contains ester bonds found in polyesters (∼10% of global plastic production), while tetradecane models the hydrocarbon chains typical of polyolefins, which account for ∼65% of plastics produced globally. Our findings therefore provide insights relevant to the two dominant plastic types worldwide.

We modified microplate-based biochemical assays to assess cell growth, cell death, redox activity (indicative of tetradecane oxidation) and esterase activity (related to DEHT hydrolysis) for the assessment of the biochemical responses of entire microbial communities over time. While these assays offer a scalable and low-cost approach for functional screening of microbial communities, they measure proxy biochemical activities, rather than directly tracking additive breakdown. Therefore, signals must be interpreted with caution, and we extended our study to include chemical analysis of terephthalic acid (TPA) production, to confirm DEHT degradation. Nonetheless, these biochemical assays allow comparative, community-level insight into microbial responses to plastic additives. We hypothesized that combining these assays with 16S rRNA amplicon sequence analysis would improve the identification of microbial consortia and species for plastic additive biodegradation. The microbial communities enriched in this study will be applied to identify biocatalysts for a novel wastewater-treatment technology developed by the BMRex consortium (www.bmrex-project.eu/) to minimize microplastic pollution.

## Materials and methods

### Collection of microbial communities from environmental plastics

Environmental samples containing mixed small plastic particles (size range 2–20 mm) were collected from a blocked stream at Swansea Bay (51.606928, −3.978028) on 1st November 2023 and from material collected by Oracle Environmental Ltd in March 2023. Chessel Bay (50.912761, −1.372012). Plastic “widgets’’ used in water treatment were collected from the primary lamella tank number 2 at Dŵr Cymru Welsh Water at Swansea Bay Waste Water Treatment Works (Wastewater Plant, 51.620224, −3.895340) on 23rd August 2023. Mixed small plastic particles (size range 2–20 mm) were collected from Swansea Council Baling Plant (Recycling Plant, 51.657794, −3.920310) on 4th January 2024, from the PET bottle sorting area. Samples were stored at room temperature, apart from the Wastewater Plant, which was stored in wastewater in a sealed plastic bottle in the fridge. All samples were stored until 27th February 2024, when the enrichment cultures were inoculated.

### Enrichment of environmental microbial communities on plastic additives

To prepare inocula, plastic pieces (5 g) of each of the four environmental samples were added to 20 mL of Tris-minimal media (Supplementary Methods) (Gorman and Levine 1965), left for 2 h, sonicated at 40 kHz for 1 min, and vortexed at 2500 rpm for 1 min. 0.14 mL of microbial inoculum of each sampling site was added to 14 mL of Tris-minimal media for enrichment (0.05 mM Tris). Cultures were supplemented with an aliquot of DEHT (0.28 mL, Fisher Scientific 10 732 202, 97% purity; final concentration: 49 mM) or tetradecane (0.28 mL, Fisher Scientific 10 317 260, 99% purity; final concentration: 75 mM). Negative control (NC) cultures were supplemented with an aliquot of Tris-minimal media, rather than substrate. Enrichment cultures were set up in 24-deep well polypropylene plates (Cambridge Biosciences 510 001) in four biological replicates. Plates were heat-sealed with QuickSeal gas permeable woven microplate film (SLS MIC9550). All plates were incubated at 200 rpm and 30°C in the dark. We ran enrichment cultures in 24-well deep-well plates to accommodate sufficient biomass while maintaining high throughput. We subsampled from the 24-well plates into 96-well plates, in which the assay measurements were taken. Four samples (1 mL each, referred to as technical replicates) were collected from each biological replicate at day 2, 7, and 22 and used to complete the assays. Samples (200 µL of the 1 mL) were centrifuged at 2250 × *g* for 10 min and cell pellets and supernatants were stored separately at −80°C for DNA sequencing and LC–MS, respectively.

### Estimation of cell abundance and viability using SYBR Green I (SGI) and propidium iodide (PI) staining

To measure DNA from all cells and dead cells, subsamples of each biological replicate (50 µL, four technical replicates) were added to a dye solution (50 µL) containing SYBR Green I (SGI; 2X final concentration, Invitrogen, Product #10 207 252), propidium iodide (PI; 5X final concentration, 7.5 µM, Acros, Product #440 300 250), or no dye in Tris-minimal media and after 5 s of orbital shaking (medium intensity), fluorescence intensity was measured (SpectraMax iD3, Molecular Devices) for 400 ms, with the photomultiplier tube (PMT) gain set to low (1 unit of optical density). The following wavelengths were used for analysis of SYBR Green I. The NC plate without dye was measured using both wavelengths, to check for auto-fluorescence.

### Measuring redox activity using the 2,6-dichlorophenolindophenol (DCPIP) assay

To assess redox activity, the method developed by Kim et al. was adapted for the study of microbial communities. Subsamples of each biological replicate (50 µL, four technical replicates) were added to clear 96-well flat-bottom, medium binding, polystyrene microplates (Greiner 655 001). The redox indicator dye 2,6-dichlorophenolindophenol (DCPIP) was added to a final concentration of 10 mg/L. Microplates were stirred at 200 rpm for 18 h at 30°C with a lid on and centrifuged for 5 min at 2250 × *g*. Optical density of the supernatant was measured at 600 nm (SPECTROstar Nano, BMG Labtech).

### Measuring esterase activity with the fluorescein dilaurate (FDL) assay

To measure esterase activity, the method developed by Liu et al. (2023) was adapted for the study of microbial communities. We found that embedding the fluorescent dye in poly(lactic acid), rather than poly(ethylene terephthalate) produced stronger esterase activity signals and modified the assay accordingly. The esterase activity assay was also modified by removing the cell lysis step such that the assay was completed using enrichment culture. Black 96-well flat-bottom, medium binding, fluotrac, polystyrene microplates (Greiner 655 076) were coated with a solution of 40 µL of 1,1,1,3,3,3-hexafluoro-2-propanol (HFIP, Sigma Aldrich 8 045 150 025) containing poly(lactic acid) mg/mL (Nanochemazone, NCZ-PA-143/24) and fluorescein dilaurate (FDL; final concentration 5 µg/mL, 7.2 µM, SLS, #46943–1G-F). Glycine buffer (50 mmol, pH 9) was added to each well of the microplate (95 µL). Subsamples of each technical replicate (5 µL, *N* = 4) were added to the filled microplate wells, which were covered with foil and incubated at 20°C for 18 h, and after 5 s of orbital shaking (medium intensity), fluorescence intensity was measured (SpectraMax iD3, Molecular Devices) for 400 ms, with the PMT gain set to low (one unit of optical density), at an excitation wavelength of 485 nm and emission wavelength of 525 nm.

### Assay data analysis

Biochemical assay plots show raw fluorescence intensity data [arbitrary units (AU)], except for the DCPIP signal, which was calculated as percentage decrease from the median NC DCPIP value (0.18). All assay data was plotted using Python v3.10.12 and the matplotlib, seaborn, pandas, numpy, scipy, and statsmodels packages (Virtanen et al. 2020). Temporal and treatment effects were assessed for each assay (SYBR Green I, PI, FDL, DCPIP) using two-sided Mann–Whitney U tests, which are robust to non-normal distributions. Pairwise comparisons were conducted (i) between sequential time points (day 7 vs day 2, day 22 vs day 7) within each substrate (Supplementary Table 1), (ii) between DEHT or tetradecane treatments and the NC at each time point (Supplementary Table 2), and (iii) within each inoculum relative to its control (Supplementary Table 3). Fold-changes were calculated as ratios of median assay values, and *P*-values were adjusted for multiple testing using the Benjamini–Hochberg false discovery rate method. Significance thresholds were set at *p*-adj ≤ .05 (*), ≤ .01 (**), ≤ .001 (***).

### LC–MS analysis

LC–MS analysis was used to measure the breakdown product TPA after hydrolysis of both ester bonds in DEHT. Samples (100 µL) collected at day 22 from enrichment cultures with DEHT, or no plastic additive, were thawed and mixed with ethyl acetate (200 µL). The organic fraction was isolated, dried overnight in a fume cupboard and mixed with 90:10 methanol: water with 0.1% formic acid (200 µL). Extracts were filtered through a PTFE syringe filter (0.22 um, Fisher Scientific 15 181 489) prior to MS analysis. MS was completed using a Dionex Ultimate 3000 HPLC system with Thermo LTQ Orbitrap XL (negative ion mode), full details are provided in the Supplementary Information. While actual TPA concentrations were beyond the remit of this study, relative quantitation of a titration of TPA was used to provide a rough estimate of differences in the relative levels of TPA measured in each enrichment cultures. The lower limit of detection of TPA was 100 ng/µL. Three biological replicates of each NC (buffer) and DEHT enrichment culture at day 22 were studied by LC–MS.

### 16S rRNA amplicon sequencing

To identify bacterial taxa in each enrichment culture, amplicon sequence analysis was completed on cell pellets from day 22. After thawing the cell pellets, the prepGEM universal kit (MicroGEM) was used for DNA extraction using the standard protocol in a 96-well PCR plate. Two PCR reactions were then completed in 96-well PCR plates, the first to amplify the variable V3 and V4 regions of the 16S rRNA gene and the second to attach index primers to the amplicons. PCRs were also completed on four positive (2 µL, 20 ng/µL, ZymoBIOMICS Microbial Community DNA Standard, D6306) and seven negative (2 µL, nuclease-free water) controls. In the first PCR, DNA template (2 µL) was added to a master-mix (18 µL) containing Platinum II Taq (10 µL, Fisher Scientific, 17 126 098), forward and reverse primers (0.4 µL, 1 µM) and nuclease-free water (7.2 µL). Primer sequences were 16S_515F: TCGTCGGCAGCGTCAGATGTGTATAAGAGACAGGTGCCAGCMGCCGCGGTAA and 16S_806R: GTCTCGTGGGCTCGGAGATGTGTATAAGAGACAGGGACTACHVGGGTWTCTAAT. In the second PCR, DNA template (2 µL) from the first PCR was added to Platinum II Taq (10 µL, Fisher Scientific, 17 126 098), two index primers (N7XX and S5XX) from the Nextera XT Index kit v2 (1 µL of each, Set B, FC-131–2002, or Set C, FC-131–2003, Supplementary Information), and nuclease-free water (7.5 µL). Both PCRs had an initial denaturation step of 95°C for 3 min followed by 30 cycles (PCR 1), or 8 cycles (PCR 2) of denaturation at 95°C for 30 s, annealing at 55°C for 30 s and extension at 72°C for 30 s. A final extension at 72°C for 5 min was completed, before cooling to 4°C. Amplicons were checked by gel electrophoresis and pooled (2 µL per sample) into those with no, weak, and three strong gel electrophoresis bands (since most samples were in the latter category). The pools were combined to give an equal concentration of each pool in the final sample. The combined pool was mixed with AMPure XP beads, cleaned with 70% ethanol (0.5 mL), and eluted in nuclease-free water (0.5 mL). After a final cleaning and concentration step, the full library was sequenced on an Illumina MiSeq platform using V3 chemistry to yield paired end reads.

### 16S amplicon sequencing data quality control and analysis

The data analysis pipeline for all samples was based on R v4.4.2 with the packages Biostrings v2.74.1, ShortRead v1.64.0, and dada2 v1.34.0 (Ihaka and Gentleman 1996, Morgan et al. 2009, Callahan et al. 2016, Lifschitz et al. 2022). Primers and adapters were trimmed with the software CUTADAPT (Martin 2011). Reads where primers were not removed, and reads shorter than 60 nucleotides were discarded; reads matching the phiX genome were removed. The dada2 library function filterAndTrim was used to remove poor quality reads with any Ns, truncate reads when quality ≤ 2, and those (after truncation) having more than two expected errors. After modeling the error rates and dereplicating identical reads, amplicon sequence variants (ASVs) were inferred using dada2, paired-end reads were merged and used to create a sequence table before removing any chimeric sequences using the removeBimeraDenovo command with default settings. Taxonomy and species were assigned using the databases “silva_nr99_v138.1_train_set.fa.gz,” and “silva_species_assignment_v138.1.fa.gz,” respectively (Quast et al. 2013). Assignments of ASVs as one of multiple possible species within a genus were permitted. ASV data were analysed using R v4.4.2 (Ihaka and Gentleman 1996) with the packages phyloseq v1.50.0, vegan v2.6.8, ggplot2 v3.5.1, microViz v0.12.6, and ANCOMBC v2.8.1 (Dixon 2003, Wickham 2011, McMurdie and Holmes 2013, Barnett et al. 2021). The R package MiscMetabar (Taudière 2023) was used to perform PERMANOVA pairwise comparisons and *P*-values were adjusted with the Bonferroni correction. Code used to generate figures was drafted using generative artificial intelligence (ChatGPT 2025), reviewed and edited, and has been deposited in the OSF database at: https://doi.org/10.17605/OSF.IO/ZKV2A.

## Results

### Plastic additives elicit recurrent biochemical responses from diverse microbial inocula

**Figure 1:**
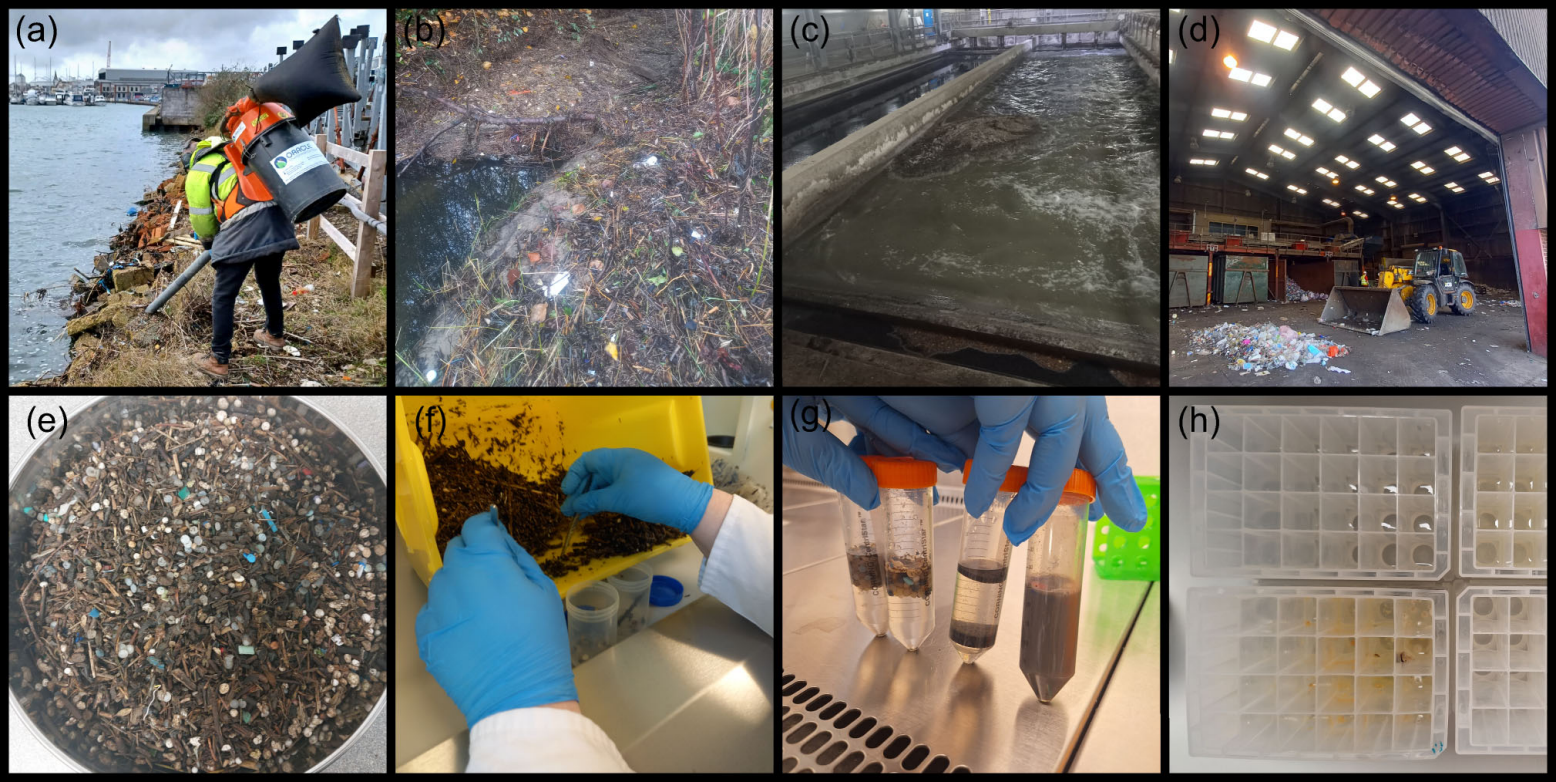
Setting up enrichment cultures with environmental microbial communities growing on plastic additives. (a–d) Environmental sampling locations. (Chessel Bay, Swansea Bay, Wastewater Plant, and Recycling Plant from left to right). (e–h) Sampling, sorting, collecting, and culturing microbial inocula in deep-well microplate enrichment cultures. (g) Inocula in same order (left to right) as (a)–(d), after addition of buffer to sorted plastic particles.

Environmental samples containing plastic debris were collected from Swansea Bay, Chessel Bay, a Recycling Plant and a wastewater treatment plant (Fig. 1a–d). Plastic particles were isolated and the associated microbial communities were collected in buffer and used to inoculate enrichment cultures in deep-well microplates (Fig. 1e–h). Enrichment cultures were established with a NC and two different plastic additives: DEHT and tetradecane.

**Figure 2:**
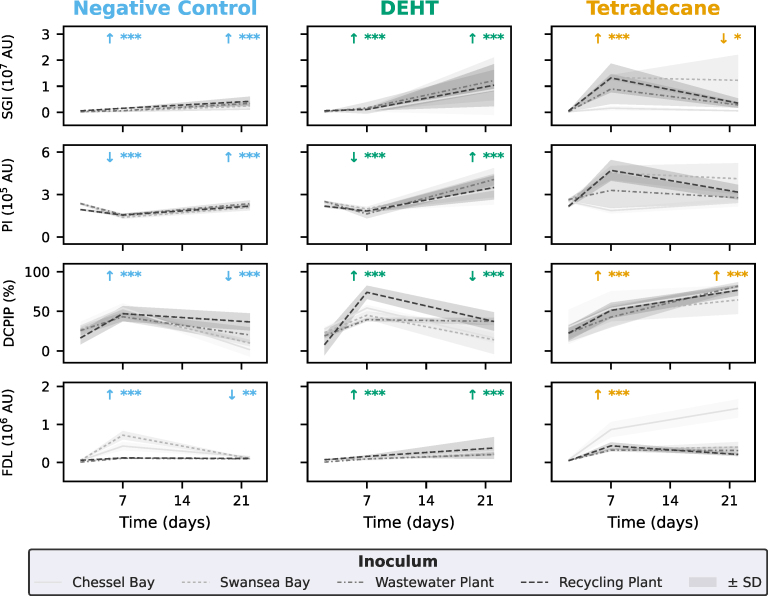
Time-course profiles of four biochemical assays measured across substrate treatments and inoculum sources. Lines represent mean values (*N* = 4) for each inoculum (see legend), for each substrate (columns: NC, DEHT, tetradecane) at day 2, 7, and 22, shaded areas indicate ± standard deviation (SD). Each row shows a different assay: total biomass (SYBR Green I, SGI assay in AU), dead cells (PI assay in AU), redox activity (2,6-dichlorophenolindophenol, DCPIP assay in %), and esterase activity (Fluoroscein dilaurate, FDL assay with PLA in AU). Temporal trends for each substrate in each assay between sequential sampling points (days 2–7 and 7–22) are indicated by arrows (↑ increase, ↓ decrease) and significance markers (* for *P* < .05, ** for *P* < .01, and *** for *P* < .001), based on pairwise comparisons across all inocula, adjusted for multiple testing.

The enrichment cultures were tested in four biochemical assays, assessing total and dead biomass, redox activity and esterase activity, 2, 7, and 22 days after inoculation (Fig. 2). Assay data revealed temporal (Supplementary Table 1) and substrate-specific Supplementary Table 2) differences in microbial activity and biomass across the 22-day incubation period, despite some variation between biological replicates (Supplementary Fig. 1). There was a significant correlation between the cell biomass and cell death assays at day 22 (*R*^2^ = 0.82), however, there was no correlation between these assays and the redox or esterase assay (Supplementary Fig. 2). The redox and esterase assays did not show a strong correlation either.

SYBR Green I (SGI), a cell-permeable fluorescent stain was used to approximate changes in cell biomass. In the NC samples, SGI fluorescence increased steadily, indicating gradual biomass accumulation. PI, a cell-impermeable stain was used to approximate changes in dead cells. For the NCs, dead cell fluorescence showed an initial decrease from day 2 to day 7, followed by a rise by day 22 in the control microbial community. Redox activity, measured by monitoring the reduction of the dye DCPIP, showed an increase in redox activity from day 2 to day 7 (*P* < .001), followed by a decrease from day 7 to day 22 (*P* < .001), for all inocula without plastic additives (NC). Meanwhile, control activity in the esterase activity assay (FDL) was split: two inocula (Chessel Bay and Swansea Bay) showed initial increases in activity from day 2 to day 7 (*P* < .001), followed by a decrease (*P* < .001). Meanwhile the Wastewater Plant and Recycling Plant showed little change in esterase activity over the 22 days.

Enrichment on DEHT resulted in a sustained and significant increase in SGI fluorescence over time for all inocula (*P* < .001), indicating continued cell proliferation. For DEHT, the cell biomass signal was only significantly higher (3.6-fold, *P* < .001) than the NC at day 22. Changes in dead cells followed a biphasic pattern similar to the control. The esterase assay also showed a steady increase over time in DEHT enrichment cultures and similarly to biomass, it was only significantly higher than the NC at day 22 (2.2-fold, *P* < .001). Redox activity increased modestly by day 7 before falling by day 22; redox activity remained active but did not scale linearly with biomass accumulation. At day 7, the Recycling Plant enriched with DEHT showed the highest redox activity, though this fell by day 22.

In contrast to DEHT, tetradecane treatments displayed a sharp, transient rise in cell biomass between days 2 and 7 (median 40-fold increase across all inocula, *P* < .001), followed by a significant decline by day 22 (*P* < .05). Dead cell measurements under tetradecane did not show significant trends between timepoints across inocula. Redox activity showed a steady and significant increase (*P* < .001) for tetradecane enrichment cultures and at day 22, this was significantly higher (79% (SD 11%), 6.3-fold higher, *P* < .001) than the NC. Esterase activity increased moderately at day 7, but declined by day 22 for all except the Chessel Bay inocula, which continued to increase, with three-fold higher median activity at day 22 than any other enrichment culture.

### DEHT was hydrolyzed into TPA

To confirm metabolism of DEHT, a qualitative LC–MS study of possible breakdown products was completed on ethyl acetate extracts from enrichment culture supernatants at day 22 (Supplementary Fig. 3A). Only the enrichment culture from the Wastewater Plant showed significant levels of TPA in all biological replicates. The NC enrichment cultures showed no TPA production. Reviewing the biochemical assay results, the Wastewater Plant enrichment cultures also demonstrated increased redox and esterase activity relative to the NC (*P* < .01) (Supplementary Table 3). A calibration curve was used to estimate that over 12% of the initial DEHT was converted to TPA in that sample (Supplementary Fig. 3B). Attempts to study other breakdown products (2-ethyl hexanol, ethylene glycol) and the starting material (DEHT) were not successful and since tetradecane is nonpolar, metabolism of this plastic additive could not be studied directly by LC–MS. In summary, LC–MS analysis of enrichment cultures was able to confirm the relative level of one DEHT biodegradation product and one enrichment culture produced TPA at day 22.

### Plastic additives enrich unique bacterial communities from the same environmental inoculum

**Figure 3:**
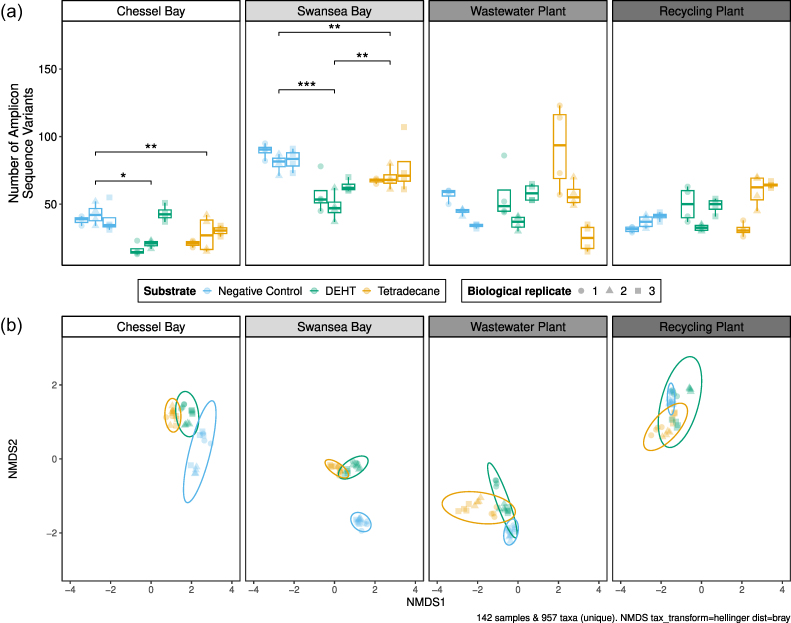
Diversity of enriched microbial communities is shaped by environmental inocula and substrate. (a) Alpha diversity (Chao1) of microbial communities across inocula and substrates. Boxplots show the estimated number of ASVs for each inoculum and substrate combination; there are three biological replicates for each combination, each of which was prepared and sequenced four times (four technical replicates). Facets correspond to different inoculum sources. Asterisks indicate pairwise Wilcoxon test significance levels between substrates within each inoculum (*P < .05, **P < .01, ***P < .001; BH-adjusted*). Error bars show interquartile ranges, with the midline representing the median. (b) NMDS using Bray–Curtis distance on Hellinger transformed bacterial community structure at ASV level (957 ASVs in 142 samples). Points represent individual technical replicates (*N* = 4) for each biological replicate (*N* = 3). Data for each inocula are shown separately, on the same axes, colored by substrate. Ellipses indicate 95% confidence intervals for each substrate group within an inoculum.

A total of 2 439 971 paired-end sequencing reads for 144 PCR samples (excluding controls) were collected after quality control, producing 957 16S rRNA ASVs. Seven NC PCRs were completed, without any DNA template, which yielded the following numbers of sequencing reads: 101, 246, 282, 343, 731, 1414, and 1613. Positive control data showed that PCRs reproduced species distributions well (Supplementary Fig. 4). For the enrichment cultures at day 22, sequencing reads for each technical replicate ranged from 0 to 69 502 reads (Supplementary Table 2). A threshold of 1000 sequencing reads was set for technical replicates and two samples were removed: Chessel Bay (biological replicate 1, technical replicate 1), which had no reads and Swansea Bay (biological replicate 1, technical replicate 2), which had 793 reads. The samples at the 10th and 90th percentiles had 5805 and 40 237 sequencing reads, respectively. Sequencing depth was adequate for recovery of the dominant species from each sample (Supplementary Fig. 5). Technical replicates were highly reproducible in terms of alpha and beta diversity (Fig. 3a, b) and so we merged all technical replicate sequencing reads for each of the 36 biological replicates for analyses of enriched species. After combining technical replicate data, the number of sequencing reads collected for each sample ranged from 9537 to 228 459 reads.

Microbial communities from natural environments (Chessel Bay, Swansea Bay) resulted in significantly less ASVs after enrichment with plastic additives, relative to the NC (*p-*adj = 0.04; Fig. 3a). Chao1, Shannon and Simpson measures all reproduced this significant decrease in alpha diversity after enrichment with DEHT and tetradecane with Bay inocula (Supplementary Fig. 6). Enrichment from anthropogenic sources (Wastewater Plant, Recycling Plant) did not lead to statistically significant changes in Chao1 Alpha diversity when the biological replicates were considered together. The Swansea Bay inocula had the highest median number of ASVs per substrate. However, the most diverse enrichment culture resulted from the Wastewater Plant inoculum with tetradecane (biological replicate 1), which had over five-fold more ASVs compared to the least diverse (Chessel Bay, DEHT, biological replicate 1) enrichment culture (158 vs 27 taxa, median of technical replicates).

Nonmetric multidimensional scaling (NMDS) based on Bray–Curtis dissimilarities of Hellinger-transformed ASV abundances produced a stable two-dimensional solution (stress = 0.097) after 20 random starts, indicating a good representation of community differences among samples. NMDS groupings by inoculant could be distinguished and broadly fall into four distinct quadrants of the NMDS1 and NMDS2 parameters, around the point (0,0) (Fig. 3b). NMDS groupings by substrate further distinguish NMDS groupings by inoculant. The dispersion of bacterial communities in NMDS space differed after enrichment with different substrates, with larger ellipses observed for DEHT and tetradecane compared with the NC for the Wastewater Plant and Recycling Plant enrichment cultures. This indicates a greater within-group variability in community composition following exposure to these substrates. To the contrary, the ellipsis for the NC from Chessel Bay culture exceeded that of the DEHT and tetradecane cultures, while for Swansea Bay, the ellipses for each treatment was similar.

To confirm the influence of substrate and inoculum on microbial diversity after enrichment, a PERMANOVA analysis was performed on a Hellinger transformation of the ASV dataset using the Bray–Curtis dissimilarity matrix with 1000 permutations. For enrichments from each inoculum, the same PERMANOVA analysis with substrate alone explained 49%, 63%, 39%, and 43% of the variance for Chessel Bay, Swansea Bay, Wastewater Plant and Recycling Plant, respectively (*P* < .001 in all cases). Meanwhile, for samples grouped by enrichment with buffer (NC), DEHT and tetradecane, the inocula explain 71%, 53%, and 52% of the variance in biodiversity respectively (*P* < .001 in all cases). Across the whole dataset, PERMANOVA showed that substrate explains 11% of the variance in biodiversity (*P* < .001), meanwhile inoculant explains 22% of the variance in biodiversity (*P* < .001).

### Plastic additives enrich known plastic-degrading families and new candidates

**Figure 4:**
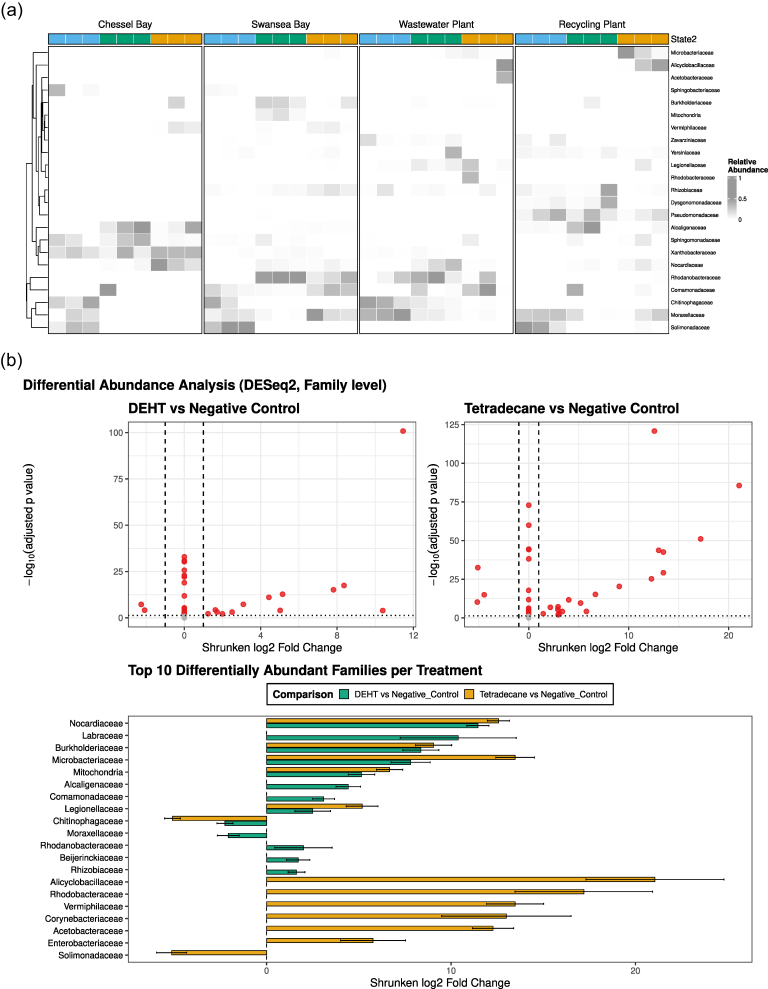
Taxonomic distribution of microbial communities growing on plastic additives. (a) Heatmap of taxonomic family abundances for each combination of inocula and substrate (State2, NC = blue, tetradecane = yellow, DEHT = green), for each biological replicate (*N* = 3). Only families with > 0.5% abundance are shown and taxa were ordered using Bray–Curtis dissimilarity-based hierarchical seriation at the family level. Color intensity (white to dark gray) represents increasing relative abundance. (b) Volcano plots show log₂-fold changes in relative abundance for each family following enrichment on DEHT (left) or tetradecane (right) compared with the NC, as determined by DESeq2 analysis of family-level counts. Each point represents a bacterial family; red points indicate significantly differentially abundant families (adjusted *P* < .05), while grey points are nonsignificant. Horizontal dotted lines mark the significance threshold (adjusted *P* = .05), and vertical dashed lines indicate two-fold change boundaries (|log₂FC| = 1). (c) Top 20 families with the largest absolute fold changes for DEHT (green) and tetradecane (orange) enrichments vs NC. Shrunken log₂-fold changes (± SE) as calculated by DESeq2 are shown. Bars correspond to mean abundance shifts relative to the NC. All log₂FC values are significant (*P* < .001).

At day 22, the samples comprised 957 ASVs, classified into 378 genera, 184 families, 112 orders, 48 classes, and 23 phyla. 19 families had an abundance of >1% of all sequencing reads. At the high taxonomical rank of class-level, bacteria with an abundance of greater than 1% were grouped into Gammaproteobacteria (13%), Alphaproteobacteria (3%), Bacteroidia (2%), Actinobacteria (2%), and Bacilli (1%). At the order level, orders with an abundance of greater than 1% included Pseudomonadales (4%), Burkholderiales (3%), Xanthomonadales (3%), Rhizobiales (1%), Chitinophagales (2%), Salinisphaerales (1%), Corynebacteriales (1%), and Alicyclobacillales (1%). Taxonomic distributions varied depending on the substrate, inocula, and biological replicate (Fig. 4a). In order of abundance, the most abundant bacterial families for each substrate and inoculum accounting for 20% of sequencing reads for each substrate-inoculum combination are outlined, in order to give an overview of the most common species for each combination. Enrichment cultures without a plastic additive (NC) led to bacterial communities where Moraxellaceae (9%), Chitinophagaceae (9%), and Solimonadaceae (8%) were most abundant. DEHT enrichment cultures were dominated by Rhodanobacteraceae (9%), Comamonadaceae (7%), and Alcaligenaceae (6%). Tetradecane enrichment cultures were dominated by Comamonadaceae (5%), Alicyclobacillaceae (5%), Moraxellaceae (4%), Nocardiaceae (4%), and Xanthobacteraceae (5%).

Each inocula resulted in enrichment of particular families with each substrate. The most abundant families after enrichment of the Chessel Bay inoculum with buffer, DEHT and tetradecane were Chitinophagaceae (3%), Alcaligenaceae (3%), and Xanthobacteraceae (3%), respectively. The most abundant families after enrichment of the Swansea Bay inoculum with buffer, DEHT, and tetradecane were Chitinophagaceae (3%), Rhodanobacteraceae (5%), and Moraxellaceae (3%), respectively. The most abundant families after enrichment of the Wastewater Plant inoculum with buffer, DEHT and tetradecane were Moraxellaceae (4%), Rhodanobacteraceae (3%), and Comamonadaceae (3%), respectively. The most abundant families after enrichment of the Recycling Plant inoculum with buffer, DEHT, and tetradecane were Solimonadaceae (4%), Alcaligenaceae (3%), and Microbacteriaceae (3%), respectively.

Differential abundance analysis revealed the bacterial families that were enriched after growth with each substrate relative to cultivation without a substrate (Fig. 4b). *Nocardiaceae, Burkholderiaceae, Microbacteriaceae*, and *Legionellaceae* were enriched on both DEHT and tetradecane, while Chitonophagaceae were less abundant on both substrates. Some families were only enriched with DEHT (and not tetradecane): Labraceae, *Alcaligenaceae, Comamonadaceae, Rhodanobacteraceae, Beijerinckiaceae*, and *Rhizobiaceae*. Other families were only enriched on tetradecane: *Alicyclobacillaceae, Rhodobacteraceae, Vermiphilaceae, Corynebacteriaceae, Acetobacteraceae*, and *Enterobacteraceae*. Of the 17 families enriched on DEHT or tetradecane, 10 have species listed as plastic-degraders in the plasticDB database (Virtanen et al. 2020), while *Laberaceae, Legionellaceae, Rhodanobacteraceae, Beijrinckiaceae, Alicyclobacillaceae, Rhodobacteraceae*, and *Vermiphilaceae* currently have no genera listed in plasticDB.

In total, 30 841 and 7163 sequencing reads were assigned to mitochondria and chloroplasts across the dataset, respectively. Mitochondria were only enriched with DEHT for the Swansea Bay inoculum [6% (SD 4%, *N* = 12) of reads] and one ASV accounted for 99% of these mitochondrial rRNAs, with a 100% match to *Andalucia godoyi* strain ATCC PRA-185 mitochondrion, which is a *Eukaryote* in the order *Jakobida*. Meanwhile, mitochondrial rRNA were enriched on tetradecane after enrichment with Chessel Bay [5% (SD 5%, *N* = 12) of reads], Swansea Bay [1% (SD 2%, *N* = 12) of reads] and Recycling Plant [5% (SD 9%, *N* = 12) of reads] inocula, with the main ASV being the *Purpureocillium lilacinum* mitochondrion, *Penicillium canescens* mitochondrion, and an uncultured organism mitochondrion closely related to mitochondria in the *Penicillium* genus, respectively. Three chloroplast rRNA ASVs were identified, though only one had >10 sequencing reads, belonging to the *Prototheca* genus, with 100% coverage and sequence identity to strains *Prototheca zopfii, Prototheca bovis*, and *Prototheca ciferri*. This nonphotosynthetic algae was found only in the Wastewater Plant enriched on tetradecane (biological replicate 1), where it accounted for 13% (SD 6%, *N* = 4) of sequencing reads. 55 mitochondrial ASVs were identified and these occurred only in samples with plastic additives (Supplementary Fig. 7).

### Biochemical activities correlate with different microbial groups

**Figure 5:**
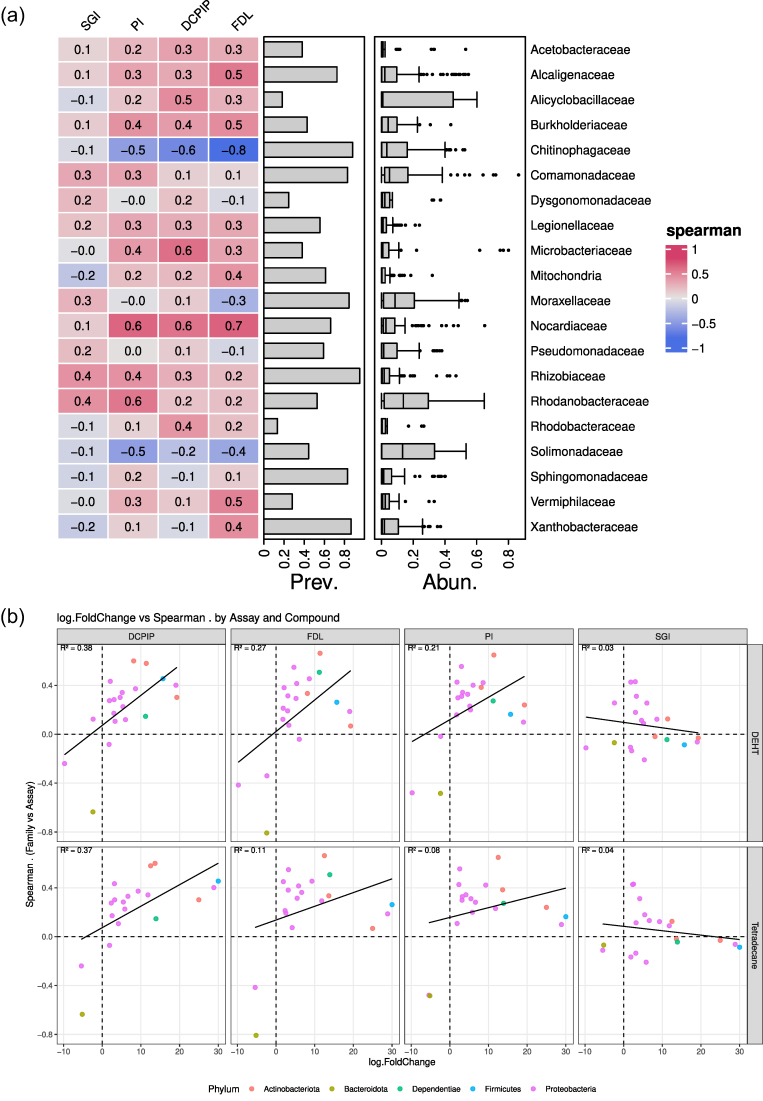
(a) Spearman correlation coefficients of relative abundance of bacterial families with assay measurements of cell biomass (SGI) and cell death (PI), redox activity (DCPIP), and esterase activity (FDL). Only bacterial families with > 0.5% abundance are shown. Red (Spearman’s *ρ* = 1) indicates a positive and blue (Spearman’s *ρ* = –1) indicates a negative correlation between assay data and taxa abundance. Prevalence (Prev.) of each family is plotted, indicating the proportion of samples in which that family was found. Abundance (Abun.) shows a bar plot of the proportion of sequencing reads matching taxa from that family across all samples. (b) Scatterplots show the relationship between log2 fold change from DESeq2 differential abundance analysis and the corresponding Spearman correlation coefficient (ρ) between each bacterial family’s relative abundance and the indicated assay variable (SGI, PI, DCPIP, or FDL). Each point represents a bacterial family colored by its phylum-level taxonomy, with panels faceted by compound (rows: DEHT and Tetradecane) and assay (columns). Dashed lines mark zero values for log2 fold change and correlation, while solid black lines indicate linear regression fits. *R*² values in each panel represent the proportion of variance in correlation explained by log2 fold change. This figure illustrates how taxonomic responses to hydrocarbon exposure relate to functional assay responses across microbial families.

We investigated patterns linking microbial community composition to functional responses. Cell biomass, cell death, redox, and esterase activity (measured with the dyes SGI, PI, DCPIP, and FDL, respectively) for all enrichment cultures at day 22 were used to calculate Pearson correlations with the bacterial families identified by microbial diversity analysis (Fig. 5a). The correlation heatmap demonstrates that several bacterial families show strong positive or negative associations with specific assay variables, suggesting that shifts in these taxa are closely tied to the physiological or metabolic activity captured by the assays. *Chitinophagaceae*, which was significantly less abundant after enrichment with DEHT and tetradecane, showed a strong negative correlation (Spearman’s *ρ* = –0.8) with the esterase assay and the redox assay (Spearman’s *ρ* = –0.6). Conversely, *Nocardiaceae*, which are abundant after enrichment with both DEHT and tetradecane, showed a strong positive correlation with the esterase assay (Spearman’s *ρ* = 0.7) and a moderate positive correlation with the redox assay (Spearman’s *ρ* = 0.6). Meanwhile, *Burkholderiaceae*, which are abundant after enrichment with both DEHT and tetradecane, showed a moderate positive correlation with esterase activity (Spearman’s *ρ* = 0.5), as did *Alcaligenaceae* and *Vermiphiliaceae*, which are enriched on DEHT and tetradecane, respectively. Correlations with the assays for cell biomass and cell death were generally weak, however a moderate positive correlation was calculated for *Rhodanobacteraceae* and *Rhizobiaceae* for both assays (Spearman’s *ρ* = 0.4–0.6).

We investigated how changes in the relative abundance of bacterial families (log₂ fold change) relate to their correlation strength with biochemical assay signals (Fig. 5b). For both DEHT and tetradecane, families that became more abundant under additive exposure showed positive correlations with assays measuring esterase (*R*² = 0.27 and 0.11, respectively) and redox activity (*R*² = 0.38 and 0.37, respectively). Notably, DEHT elicited a stronger correlation of esterase activity with enrichment than tetradecane, while bacterial families enriched by both substrates had enhanced correlations with redox activity. Correlations with the cell viability assays (SGI and PI) were weaker (*R*² ≤ 0.10), apart from the cell death assay with DEHT (*R*² = 0.21), consistent with the idea that growth and death responses were distributed more evenly across the community.

## Discussion

Plastic pollution releases a wide range of chemical additives into the environment, many of which are bioavailable and potentially toxic. While the microbial degradation of plastic polymers has been studied extensively, the ecological and functional responses of microbial communities to plastic additives themselves remain poorly understood (Oberbeckmann and Labrenz 2020). To address this gap, we developed a high-throughput screening platform that integrates biochemical assays and 16S rRNA amplicon sequencing to assess the metabolism of plastic additives by entire microbial consortia. This community-level perspective allows direct measurement of functional activity rather than inference from gene presence alone.

We applied this framework to four environmental inocula differing in exposure history: two natural environments (Chessel Bay and Swansea Bay) experiencing intermittent plastic contamination, and two anthropogenic environments (a Wastewater Plant and a recycling facility) subjected to chronic plastic exposure. This design enabled comparison of microbiomes with contrasting baseline adaptations to synthetic chemicals. Each inoculant was a microbial community taken from plastic in a particular environment, where the microbial consortia had essentially been pre-enriched. In our experiments, those pre-enriched communities were further enriched on the additive containing media. We did not characterize the environmental plastic samples and could not confirm whether they contained DEHT or tetradecane. The Recycling Plant and Wastewater Treatment Plant are likely to have contained PAE, as shown in environmental studies of similar environments (Zolfaghari et al. 2014), especially since the Recycling Plant specimen came from a PET bottle sorting area. Nonetheless, consistent cell growth of all inocula enriched with both substrates within the 22 day experiment highlights the relative ease of plastic additive biodegradation (relative to plastic degradation). This could also indicate that enzymes that degrade some plastic additives may be found in most environments, as has been shown for biodegradeable polyesters (Degli-Innocenti et al. 2023).

Marine microbiome studies have shown that plastic substrates select for distinct bacterial assemblages depending on polymer type (Hansen et al. 2021) and environmental context (Oberbeckmann et al. 2018); our results extend this concept to the level of plastic additives. In our study, inocula from anthropogenic sites retained greater alpha diversity, exhibiting broader taxonomic responses to additive enrichment, consistent with pre-adaptation to plastic-associated compounds. In contrast, communities from natural environments displayed significant declines in alpha diversity, indicating selection for specialized taxa. We also developed biochemical assays which offer a scalable method to identify microbial consortia capable of plastic additive degradation and compared these to microbial community composition.

To capture metabolic responses to plastic additives, we modified fluorescence- and absorbance-based assays to measure cell growth, death, redox activity, and esterase activity across environmental microbial communities. A variety of high-throughput strategies are available for screening plastic-degrading enzymes (Yew et al. 2025) and many focus on testing protein variants, which necessitates cell lysis and protein purification steps. These can be mitigated by tagging proteins for secretion with YebF, an 11 kDa soluble protein (Zurier and Goddard 2023), or by protein surface display (Heyde et al. 2021). However, we took a different approach—testing cell culture rather than cell lysate samples in order to monitor the activity of entire microbiomes and reduce the number of steps in each assay. We selected assays to monitor redox activity and esterase activity as these processes are likely to be implicated in polyolefin and polyester biodegradation respectively (Inderthal et al. 2021, Liu et al. 2023). The esterase assay (Liu et al. 2023) itself had been modified from integrating FDL into a film, rather than particles (Zumstein et al. 2017). Also, the dye was integrated into a PLA film, rather than PET. Fluorescence emitted following ester cleavage of FDL in PLA films by cell culture samples was monitored. Modifications were also made to the redox assay developed by Kim et al. (2023), applying it to study enrichment culture samples, rather than testing individual bacterial strains.

While biochemical assays provide a rapid and inexpensive assessment of plastic-degradative potential, they measure proxy activities that may reflect nonspecific redox or hydrolytic reactions not directly linked to additive transformation. Both false positive and false negative assay results are possible. For instance, the redox assay using DCPIP could give a false positive response for microbial metabolic stress, production of nonspecific electron donors, or redox-active metabolites unrelated to DEHT or tetradecane degradation. Similarly, the esterase assay may detect general hydrolytic enzymes that act on PLA film and the FDL dye, producing elevated fluorescence and is not necessarily indicative of DEHT degradation. Conversely, active degradation of the additives may go undetected if the esterase pathway does not interact with the assay substrate, or if metabolic activity is below detection thresholds. Therefore, direct linkage of gene expression and metabolite profiles would be needed to confirm degradation pathways. Future work should include LC–MS or GC–MS to track disappearance of parent compounds and formation of intermediates, alongside isotope labeling to unambiguously trace carbon flow from additives into microbial biomass or metabolites. Moreover, longer incubation periods could uncover slow-growing taxa or cooperative interactions not captured within 22 days.

In pre-experiments, we found that microplastic substrates can interfere in the fluorescence and absorbance measurements involved in the assays, likely due to light scattering, autofluorescence, and nonspecific dye adsorption. Due to their crystalline structure, plastics are often slower to biodegrade than plastic additives, which are noncrystalline. Plastic additives are often liquids, making them easier to handle in high-throughput microplate-based assays and less likely to interfere with them. An experiment studying lactic acid biodegradation was also completed, however it interfered with the assays, reducing the signal to noise ratios to impractical levels (data not shown). To mitigate interference from microplastics, all enrichment experiments were performed using plastic additive substrates only (DEHT and tetradecane), without added microplastic particles, ensuring that the observed biochemical signals reflected microbial responses to dissolved additives rather than plastic particles.

This study demonstrates distinct microbial community responses to the plasticizer DEHT and the hydrocarbon tetradecane, revealed through time-resolved biochemical assays tracking cell biomass, dead cells, esterase activity, and redox potential. Distinct biochemical responses were observed for each additive. Specifically, cell growth, death, redox activity and esterase activity were measured after 2, 7, and 22 days of enrichment on two different plastic additives. The strong correlation between total and dead cell fluorescence signals suggests that overall microbial biomass production was accompanied by increased cell turnover, a pattern characteristic of active microbial communities under substrate stress. Oil-degrading marine consortia show rapid hydrocarbon oxidation, followed by nutrient exhaustion and cell turnover (Head et al. 2006, Yu et al. 2022). On the other hand, the redox and esterase signals did not have a strong correlation, suggesting that they represent distinct biochemical functions. Furthermore, while there was a significant increase for all enrichment cultures from day 2 to day 7, the trends from day 7 to day 22 differed for tetradecane and DEHT, suggesting that the two additives engage largely independent metabolic pathways and potentially distinct microbial communities.

Substrate-specific biochemical response dynamics were generally consistent across inocula, suggesting convergent functional responses of the diverse enrichment cultures. Some cellular growth was observed on the NC after 22 days in concordance with a recent publication, showing that Tris can be used by bacteria as a carbon source (Holert et al. 2024). Tetradecane was metabolized faster than DEHT, with cell biomass peaking at day 7, rather than day 22. Nonetheless, the maximum cellular biomass signal was similar for both substrates. Median cellular growth with tetradecane decreased from day 7 to day 22 for enrichment cultures from all inocula, while for DEHT, it is possible that cellular growth could have continued past day 22. This is consistent with previous studies of biodegradation of these substrates by environmental inocula, where at 7 days, 78% of long-chain alkanes were degraded (Yu et al. 2022), compared to 50% of DEHP (a structural isomer of DEHT) (Saeng-kla et al. 2025).

In contrast, microbial growth in response to tetradecane was transient. The sharp rise in SYBR fluorescence between days 2 and 7 followed by a decline by day 22 could reflect rapid early proliferation of hydrocarbon-degrading taxa, a common feature in long-chain alkane metabolism (Yu et al. 2022). The concurrent decline in esterase activity could support this interpretation, indicating reduced esterase activity once the primary substrate pool was depleted, though redox activity continued to increase. This boom-and-bust pattern aligns with hydrocarbon bioremediation models in which fast-growing degraders dominate early but are later replaced by slower-growing, generalist taxa as energy yields decline (Head et al. 2006).

Redox activity was highest for tetradecane, which could be because oxidation is the first step in alkane biodegradation. Redox activity for DEHT peaked at day 7, before falling and this early redox activity could be associated with oxidation of DEHT hydrolysis products, such as dioxygenolysis of TPA (Salvador et al. 2019). However, redox activity of DEHT enrichment cultures was not significantly different to the NC at any timepoint. The strong and sustained increases in cell biomass and esterase activity under DEHT exposure suggest that the microbial consortia not only tolerated DEHT but may have utilized it or its breakdown products as carbon or energy sources, consistent with phthalate analogue degradation (Paluselli et al. 2018). Chessel Bay enrichment cultures with tetradecane showed exceptional esterase activity, which may be due to oxidation of the long-chain alkane to form an ester and subsequent hydrolysis (Ji et al. 2013). This finding illustrates the utility of these assays to identify microbial consortia with a propensity for biodegradation of particular substrates. The plastic additives that we studied have chemical functional groups found in many plasticizers and therefore our methodology could be scaled up to study large combinations of plastic additives and diverse environmental inocula.

Taken together, the assay data reveal a broader ecological contrast between DEHT and tetradecane-enriched conditions, with each emphasizing different hallmarks of plastic biodegradation. DEHT exposure favors sustained esterase activity and biomass growth, while tetradecane elicits rapid, but transient growth and long-term redox-activity. These differences are consistent with observations that hydrocarbon oxidation and ester hydrolysis are catalyzed by different enzymatic functions. Redox activity, measured through DCPIP reduction, primarily reflects oxidative metabolism such as alkane oxidation and the production of ROS, whereas esterase activity indicates hydrolysis of ester bonds as occurs in the breakdown of phthalates or polyesters. This distinction underscores how different xenobiotic substrates shape microbial succession and metabolic diversity. These findings indicate that plastic additives can act as ecological filters, selectively enriching bacterial taxa capable of both tolerance and transformation of both ester- and alkane-based plasticizers and plastic polymers.

LC–MS was used to investigate degradation of DEHT, by monitoring production of the breakdown product TPA. Only the enrichment cultures inoculated from the Wastewater Plant had quantifiable levels of TPA, equivalent to a minimum of 12% conversion of the substrate after 22 days. The absence of TPA in other enrichment cultures does not rule out DEHT metabolism as the produced TPA could have been metabolized to other products. TPA is often metabolized into protocatechuic acid, which is utilized for carbon and energy (Salvador et al. 2019). While it is theoretically possible that TPA could arise from alternative metabolic processes unrelated to DEHT, such as from environmental polyester contaminants or endogenous aromatic compounds, DEHT was the only added carbon source in these cultures, and NCs with DEHT omitted showed no TPA formation. Therefore, under the specific conditions of this experiment, DEHT biodegradation remains the most likely source of TPA. Furthermore, TPA is a highly reduced aromatic compound, whereas ambient CO₂ is a fully oxidized form of carbon. The biosynthesis of TPA from CO₂ would require substantial energy input and multiple reductive steps. To our knowledge, no microbial biosynthetic pathways have been identified that can produce TPA *de novo* from CO₂. LC–MS measurements of ambient TPA levels in community wastewater samples of 21 ± 10 µg L⁻¹ by Kumar et al. (2022) (Arizona, USA, 2022) are three orders of magnitude lower than the concentration measured in DEHT enrichment cultures, which exceeded 25 mg L⁻¹.

LC–MS was limited to a single metabolite and encountered background interference, highlighting the value of complementary untargeted metabolomics in future studies. Analysis of changes in levels of the starting material would give a more complete picture of substrate degradation. Tetradecane metabolism could not be confirmed via LC–MS due to its volatility and lack of ionizable functional groups, though GC-MS may offer more direct confirmation of alkane degradation in future work. While our LC–MS study was limited in scope, it did confirm production of significant levels of one DEHT biodegradation product by all biological replicates of one enrichment culture. Our biochemical assay platform offers a less expensive and more accessible insight into plastic biodegradation which can be applied combinatorially with different inocula and additives to identify microbial communities with high potential for finding plastic-degrading gene candidates.

16S rRNA amplicon sequencing showed that exposure to DEHT and tetradecane drives reproducible, substrate-specific changes in microbial community structure and function across inocula from a range of environments. Both substrate type and inoculum origin significantly influenced bacterial community composition. Substrate explained 39%–63% of the variance in ASV profiles within each inoculum, meanwhile inocula could explain 52%–71% of the variance for enrichment cultures grouped by substrate. This microbial community divergence is consistent with patterns after exposure to different plastics (Scales et al. 2021) and following enrichment of plastic-based substrates by different environmental inocula (Sheridan et al. 2022).

Alpha diversity declined in natural inocula (Chessel Bay, Swansea Bay) after enrichment, likely reflecting strong selective pressure by the additives. In contrast, the Alpha diversity of Wastewater Plant and Recycling Plant cultures did not change significantly, possibly due to prior exposure to plastic-associated pollutants. These patterns support the idea that environmental history—i.e. chronic vs. intermittent exposure to plastic pollution—influences microbial community structure, diversity, and functional capacity. Our study assessed bacterial composition at only one timepoint. However, Schaerer et al. showed that Alpha diversity of microbial communities from five environmental sources decreased upon enrichment with plastic additives. Consistently with our study, enrichment on the plastic metabolites terepthalate and terephthalamide resulted in a greater decrease in Alpha diversity than for the control enrichment (no additive) (Schaerer et al. 2024).

Comparisons between inocula highlight the importance of environmental provenance in determining biodegradation potential. Microbial communities from natural habitats (Chessel Bay, Swansea Bay) showed pronounced declines in alpha diversity after enrichment, implying strong selective pressure toward specialist taxa. In contrast, anthropogenic sources (Wastewater Plant, Recycling Plant) retained higher diversity and activity, suggesting functional redundancy and prior adaptation to plastic-derived pollutants. The Wastewater Plant community exhibited the highest esterase and redox activities and was the only enrichment in which DEHT hydrolysis to TPA was confirmed. These patterns support the view that wastewater environments serve as evolutionary incubators for xenobiotic-degrading microorganisms, enriched by continual exposure to industrial and domestic plastic residues (Saeng-kla et al. 2025).

Despite differences in microbial community composition, our biochemical assays showed consistent patterns for each substrate, suggesting convergence of biochemical function. These findings are consistent with research showing that plastic-degrading microorganisms are ubiquitous (Scales et al. 2021) and that plastic leachate can foster more microbial growth than natural organic matter (Sheridan et al. 2022). Therefore, we investigated which microbial families were enriched in the presence of each substrate.

Taxonomic analysis identified several bacterial families enriched by additive exposure that are well known for hydrocarbon and plastic degradation. *Nocardiaceae, Burkholderiaceae*, and *Microbacterium* dominated cultures enriched with DEHT and tetradecane. These are families whose members encode oxidases and esterases implicated in alkane and phthalate breakdown (Borre and Sonnenschein 2021, Schaerer et al. 2023). Schaerer et al. highlighted *Rhodococcus*, in the *Nocardiaceae* family, as a generalist was recently highlighted as a plastic-degrading generalist, enriched on terepthalic acid. HDPE and deconstructed PET (Schaerer et al. 2023). *Alcaligenaceae* and *Comamonadaceae* were only enriched on DEHT. Species in the *Comamonadaceae* family were enriched from plastics incubated with soil, seawater and wastewater samples (Borre and Sonnenschein 2021). Wilkes et al. showed that *Comamonas* are enriched on PET microplastics in wastewater and cause fragmentation into nanoplastics via ester hydrolysis (Wilkes et al. 2024) and were enriched on DEHP along with *Alcaligenaceae* (Kou et al. 2023).

Seven bacterial families enriched in DEHT or tetradecane treatments currently have no genera formally linked to plastic degradation in PlasticDB, the plastic biodegradation database (Gambarini et al. 2022). However, multiple studies suggest that some of these families may have relevant metabolic capacities for biodegradation. Those enriched on tetradecane, included *Vermiphiliaceae, Rhodobacteraceae*, and *Alicyclobacillaceae; Alicyclobacilaceae* were also enriched on polyethylene under aerobic conditions (Yu et al. 2022) and *Rhodobacteraceae* re-occur on marine plastic debris (Roager and Sonnenschein 2019, Erni-Cassola et al. 2020) and plastic leachates (Birnstiel et al. 2022). *Rhodanobacteraceae* were enriched on expanded polystyrene (Liu et al. 2023) and polyethylene microplastics . *Legionellaceae* are known to form biofilms and persist in environments such as plumbing systems, suggesting adaptation to carbon-limited conditions and potential resilience on plastic-associated surfaces (Ma et al. 2020). *Vermiphilaceae*, intracellular bacteria hosted by protists, though poorly characterized, have been detected in plastic-associated microbial consortia in coral reef environments (Singleton et al. 2023).

Taken together, the 17 enriched families—those with established roles in plastic degradation (e.g. *Nocardiaceae, Burkholderiaceae, Comamonadaceae, Alcaligenaceae, Rhizobiaceae*) and those with emerging or uncharacterized potential—represent a valuable reservoir for future biotechnological exploration. Families containing model organisms such as *Enterobacteraceae* may offer genes prone to expression in *Escherichia coli* and facile metabolic engineering: known esterases, monooxygenases, and dioxygenases could be repurposed for additive breakdown. At the same time, lesser-known families may encode novel catabolic pathways adapted to unique environmental niches such as wastewater biofilms, thermophilic composts, or marine plastics. Facultative taxa in the family *Rhodobacteraceae*, for instance, may enhance performance in engineered consortia by contributing niche versatility (Simon et al. 2017). By prioritizing these taxa in future shotgun metagenomic, proteomic, and transcriptomic studies, it may be possible to discover new degradation pathways for plastic additives and advance biotechnologies for bioremediating plastic and plastic additive pollution and closing material loops in circular economies.

The enrichment cultures were performed in the dark to avoid algal growth and encourage enrichment of bacteria, which would be characterized by 16S rRNA amplicon sequencing. Chloroplast rRNA from *Prototheca*, nonphotosynthetic obligate algal heterotrophs containing horizontally-transferred genes from bacteria (Jian et al. 2024), was found in additive-enriched cultures. In addition to bacterial and chloroplast rRNA, mitochondrial rRNA was enriched for both DEHT and tetradecane, associated with the fungi *Purpureocillium* and *Penicillium*. These fungi have been associated with biodegradation of polyurethane and the biodegradable polyester PBAT (Bhavsar et al. 2024, Ferreira-Filipe et al. 2024). These findings suggest auxiliary roles for fungi and algal-like eukaryotes in plastic additive transformation. Eukaryotic microorganisms are increasingly recognized for secreting esterases and monooxygenases that attack hydrophobic polymers and complex organic substrates (Temporiti et al. 2022). Their co-occurrence with bacterial degraders indicates that mixed bacterial–eukaryotic consortia may drive synergistic transformations of plastic additives. This cross-domain interaction underscores the need for integrated metagenomic analyses to elucidate the functional roles of fungi and protists in biodegradation networks (Salinas et al. 2024).

Correlation analyses between biochemical assay signals and bacterial abundance revealed functional associations. *Nocardiaceae* and *Burkholderiaceae*, known for their ability to degrade pollutants (Ma et al. 2023, Ridley et al. 2024), both showed strong positive correlations with esterase and redox activity, suggesting direct enzymatic involvement in substrate oxidation and hydrolysis. *Chitinophagaceae*, which were enriched in the NCs had a strong negative correlation with esterase and redox activity. Redox and esterase activity Spearman correlations showed a positive correlation with log-fold change for cultures enriched with both DEHT and tetradecane. Abundance of bacterial families which had higher fold-enrichment in DEHT, an ester-containing phthalate showed a stronger correlation with esterase activity than those enriched in tetradecane. Overall, the results show that ester and hydrocarbon degradation potential is unevenly distributed across the community and largely concentrated in a few taxa that respond both compositionally and functionally to chemical stress. These findings align with previous reports of cooperative metabolism in polymer-degrading consortia, where different microbial groups perform sequential steps in breakdown and mineralization (Zumstein et al. 2017, Oberbeckmann and Labrenz 2020). Integrating such functional assays with taxonomic data provides a scalable method to identify key biodegradative families in mixed microbial communities. Unlike single-isolate assays, the use of mixed communities captures the synergistic interactions and cross-feeding that often underpin effective substrate degradation.

Our results highlight how plastic additives act both as pollutants and as ecological drivers, selecting for microbiomes with appropriate catabolic versatility. By identifying taxa and enzymatic functions associated with additive metabolism, this study provides a foundation for developing biotechnological solutions that biodegrade both plastic additives and polymers. Additive-responsive communities identified here could inform the design of microbial communities for bioremediation. Integrating these naturally occurring biodegraders into bioprocesses may reduce plastic additive persistence, mitigate ecotoxicity (Zimmermann et al. 2021) and advance microbial circular-economy strategies. Future studies should integrate shotgun metagenomics, metatranscriptomics or metabolomics to identify the specific enzymes and regulatory networks underlying additive metabolism (Wright et al. 2021). The approach established here—combining high-throughput functional screening with community sequencing—provides a scalable blueprint for such efforts and can be readily adapted to other plastic additives or polymers.

## Conclusion

Overall, this study provides strong evidence that microbial genera capable of plastic degradation can also metabolize plastic additives. Across multiple environmental inocula, plastic additives such as DEHT and tetradecane elicited reproducible patterns of growth, redox, and esterase activity, highlighting consistent functional responses to distinct chemical structures. These high-throughput biochemical assays captured broad differences in microbial metabolism of ester- and alkane-based additives, demonstrating their value as scalable, low-cost tools for screening microbial communities. LC–MS analysis confirmed biodegradation of DEHT in at least one enrichment culture, validating the metabolic activity detected in the assays. Plastic additives reshaped microbial community structure: 16S rRNA sequencing revealed that enrichment on plastic additives decreased alpha diversity for some inocula and selected for bacterial taxa previously associated with plastic degradation, as well as novel candidates. These enriched bacterial families represent promising starting points for further exploration of plastic and additive biodegradation. The convergence of biochemical assay data and microbial community analysis confirms that diverse environmental microbiomes can metabolize additive components representative of major plastic classes. Moving forward, selected consortia will be applied to the development of an integrated wastewater treatment technology by the BMRex consortium (www.bmrex-project.eu).

## Supplementary Material

qvag003_Supplemental_File

## Data Availability

The raw sequencing data generated by this study along with all Supplementary Figures, Tables, Methods, and code has been deposited in the OSF database at: https://doi.org/10.17605/OSF.IO/ZKV2A.

## References

[bib1] Barnett DJ, Arts IC, Penders J. microViz: an R package for microbiome data visualization and statistics. J Open Source Softw. 2021;6:3201. 10.21105/JOSS.03201

[bib2] Bhattacharyya M, Dhar R, Basu S et al. Molecular evaluation of the metabolism of estrogenic di(2-ethylhexyl) phthalate in *Mycolicibacterium* sp. Microb Cell Fact. 2023;22:1–14. 10.1186/S12934-023-02096-0 37101185 PMC10134610

[bib3] Bhavsar P, Bhave M, Webb HK. Effective multi-stage biodegradation of commercial bulk polyurethane by *Clonostachys* and *Purpureocillium* spp. Sci Total Environ. 2024;908:168329. 10.1016/J.SCITOTENV.2023.168329 37926262

[bib84_901_101626] Birnstiel S, Sebastián M, Romera-Castillo C. Structure and activity of marine bacterial communities responding to plastic leachates. Science of The Total Environment. 2022;834:155264. 10.1016/j.scitotenv.2022.155264 35439504

[bib4] Borre I, Sonnenschein EC. Draft genome sequences of nine environmental bacterial isolates colonizing plastic. Microbiol Resour Announc. 2021;10:e01485–20. 10.1128/MRA.01485-20 33737371 PMC7975889

[bib5] Bu S, Wang Y, Wang H et al. Analysis of global commonly-used phthalates and non-dietary exposure assessment in indoor environment. Build Environ. 2020;177:106853. 10.1016/J.BUILDENV.2020.106853

[bib6] Callahan BJ, McMurdie PJ, Rosen MJ et al. DADA2: high-resolution sample inference from Illumina amplicon data. Nat Methods. 2016;13:581–3. 10.1038/NMETH.3869 27214047 PMC4927377

[bib7] Caporaso JG, Kuczynski J, Stombaugh J et al. QIIME allows analysis of high-throughput community sequencing data. Nat Methods. 2010;7:335–6. 10.1038/NMETH.F.303 20383131 PMC3156573

[bib8] Caporossi L, Alteri A, Campo G et al. Cross sectional study on exposure to BPA and phthalates and semen parameters in men attending a fertility center. Int J Environ Res Public Health. 2020;17:489. 10.3390/IJERPH17020489 31940982 PMC7013870

[bib9] Chao WL, Cheng CY. Effect of introduced phthalate-degrading bacteria on the diversity of indigenous bacterial communities during di-(2-ethylhexyl) phthalate (DEHP) degradation in a soil microcosm. Chemosphere. 2007;67:482–8. 10.1016/J.CHEMOSPHERE.2006.09.048 17092544

[bib10] ChatGPT. 2025. https://chatgpt.com/ (31 October 2025, date last accessed).

[bib11] Chen CY. Biosynthesis of di-(2-ethylhexyl) phthalate (DEHP) and di-n-butyl phthalate (DBP) from red alga—*Bangia atropurpurea*. Water Res. 2004;38:1014–8. 10.1016/J.WATRES.2003.11.029 14769421

[bib12] Chen Y, Zhen Z, Li G et al. Di-2-ethylhexyl phthalate (DEHP) degradation and microbial community change in mangrove rhizosphere gradients. Sci Total Environ. 2023;871:162022. 10.1016/J.SCITOTENV.2023.162022 36775151

[bib13] Degli-Innocenti F, Breton T, Chinaglia S et al. Microorganisms that produce enzymes active on biodegradable polyesters are ubiquitous. Biodegradation. 2023;34:489–518. 10.1007/S10532-023-10031-8 37354274

[bib14] Deyo JA. Carcinogenicity and chronic toxicity of di-2-ethylhexyl terephthalate (DEHT) following a 2-year dietary exposure in Fischer 344 rats. Food Chem Toxicol. 2008;46:990–1005. 10.1016/J.FCT.2007.10.037 18077073

[bib15] Dixon P. VEGAN, a package of R functions for community ecology. J Veg Sci. 2003;14:927–30. 10.1111/J.1654-1103.2003.TB02228.X

[bib16] Douglas GM, Maffei VJ, Zaneveld JR et al. PICRUSt2 for prediction of metagenome functions. Nat Biotechnol. 2020;38:685–8. 10.1038/S41587-020-0548-6 32483366 PMC7365738

[bib17] Erni-Cassola G, Wright RJ, Gibson MI et al. Early colonization of weathered polyethylene by distinct bacteria in marine coastal seawater. Microb Ecol. 2020;79:517–26. 10.1007/S00248-019-01424-5 31463664 PMC7176602

[bib18] Farias JDM, Argolo LA, Neves RAF et al. Mangrove consortium resistant to the emerging contaminant DEHP: composition, diversity, and ecological function of bacteria. PLoS One. 2025;20:e0320579. 10.1371/JOURNAL.PONE.0320579 40273087 PMC12021221

[bib19] Ferreira-Filipe DA, Oliveira L, Paço A et al. Biodegradation of e-waste microplastics by *Penicillium brevicompactum*. Sci Total Environ. 2024;935:173334. 10.1016/J.SCITOTENV.2024.173334 38763191

[bib20] Gambarini V, Pantos O, Kingsbury JM et al. PlasticDB: a database of microorganisms and proteins linked to plastic biodegradation. Database. 2022;2022:1–12. 10.1093/DATABASE/BAAC008 PMC9216477 35266524

[bib21] Gorman DS, Levine RP. Cytochrome f and plastocyanin: their sequence in the photosynthetic electron transport chain of *Chlamydomonas reinhardi*. Proc Natl Acad Sci USA. 1965;54:1665–9. 10.1073/PNAS.54.6.1665 4379719 PMC300531

[bib22] Hansen J, Melchiorsen J, Ciacotich N et al. Effect of polymer type on the colonization of plastic pellets by marine bacteria. FEMS Microbiol Lett. 2021;368:fnab026. 10.1093/FEMSLE/FNAB026 33640965

[bib23] Head IM, Jones DM, Röling WFM. Marine microorganisms make a meal of oil. Nat Rev Micro. 2006;4:173–82. 10.1038/NRMICRO1348 16489346

[bib24] Heyde SAH, Arnling Bååth J, Westh P et al. Surface display as a functional screening platform for detecting enzymes active on PET. Microb Cell Fact. 2021;20:1–9. 10.1186/S12934-021-01582-7 33933097 PMC8088578

[bib25] Holert J, Borker A, Nübel LL et al. Bacteria use a catabolic patchwork pathway of apparently recent origin for degradation of the synthetic buffer compound TRIS. ISME J. 2024;18:1–11. 10.1093/ISMEJO/WRAD023 PMC10848231 38365256

[bib26] Ihaka R, Gentleman R. R: a language for Data analysis and graphics. J Comput Graph Statist. 1996;5:299–314. 10.1080/10618600.1996.10474713

[bib27] Inderthal H, Tai SL, Harrison STL. Non-hydrolyzable plastics—an interdisciplinary look at plastic bio-oxidation. Trends Biotechnol. 2021;39:12–23. 10.1016/j.tibtech.2020.05.004 32487438

[bib28] Ji Y, Mao G, Wang Y et al. Structural insights into diversity and n-alkane biodegradation mechanisms of alkane hydroxylases. Front Microbiol. 2013;4:42005. 10.3389/FMICB.2013.00058 PMC3604635 23519435

[bib29] Jian J, Wang Z, Chen C et al. Two high-quality *Prototheca zopfii* genomes provide new insights into their evolution as obligate algal heterotrophs and their pathogenicity. Microbiol Spectr. 2024;12:e04148–23. 10.1128/SPECTRUM.04148-23 38940543 PMC11302234

[bib30] Khoshmanesh M, Farjadfard S, Ahmadi M et al. Review of toxicity and global distribution of phthalate acid esters in fish. Sci Total Environ. 2024;953:175966. 10.1016/J.SCITOTENV.2024.175966 39245393

[bib31] Kim D, Cui R, Moon J et al. Soil ecotoxicity study of DEHP with respect to multiple soil species. Chemosphere. 2019;216:387–95. 10.1016/j.chemosphere.2018.10.163 30384308

[bib32] Kim HR, Lee C, Shin H et al. Isolation of a polyethylene-degrading bacterium, *Acinetobacter guillouiae*, using a novel screening method based on a redox indicator. Heliyon. 2023;9:e15731. 10.1016/J.HELIYON.2023.E15731 37180881 PMC10173618

[bib33] Kou L, Chen H, Zhang X et al. Biodegradation of di(2-ethylhexyl) phthalate by a new bacterial consortium. Water Sci Technol. 2023;88:92–105. 10.2166/WST.2023.198 37452536

[bib34] Kumar R, Adhikari S, Driver E et al. Application of wastewater-based epidemiology for estimating population-wide human exposure to phthalate esters, bisphenols, and terephthalic acid. Sci Total Environ. 2022;847:157616. 10.1016/J.SCITOTENV.2022.157616 35901875

[bib35] Lamraoui I, Eltoukhy A, Wang J et al. Biodegradation of di (2-Ethylhexyl) phthalate by a novel Enterobacter spp. Strain YC-IL1 isolated from polluted soil, Mila, Algeria. Int J Environ Res Public Health. 2020;17:7501. 10.3390/IJERPH17207501 33076331 PMC7602616

[bib36] Lifschitz S, Haeusler EH, Catanho M et al. Bio-strings: a relational database data-type for dealing with large biosequences. BioTech. 2022;11:31. 10.3390/BIOTECH11030031 35997339 PMC9472027

[bib37] Liu K, Xu Z, Zhao Z et al. A dual fluorescence assay enables high-throughput screening for poly(ethylene terephthalate) hydrolases. ChemSusChem. 2023;16:e202202019. 10.1002/CSSC.202202019 36511949

[bib85_787_102626] Ma X, Pierre D, Bibby K, Stout JE. Bacterial community structure correlates with Legionella pneumophila colonization of New York City high rise building premises plumbing systems. Environmental Science: Water Research & Technology. 2020;6:1324–1335. https://pubs.rsc.org/en/content/articlehtml/2020/ew/c9ew01084j

[bib38] Ma Y, Wang J, Liu Y et al. Nocardioides: “specialists” for hard-to-degrade pollutants in the environment. Molecules. 2023;28:7433. 10.3390/MOLECULES28217433 37959852 PMC10649934

[bib39] Martens-Habbena W, Sass H. Sensitive determination of microbial growth by nucleic acid staining in aqueous suspension. Appl Environ Microb. 2006;72:87. 10.1128/AEM.72.1.87-95.2006 PMC1352258 16391029

[bib40] Martin M. Cutadapt removes adapter sequences from high-throughput sequencing reads. EMBnet J. 2011;17:10–2. 10.14806/EJ.17.1.200

[bib41] McMurdie PJ, Holmes S. phyloseq: an R package for reproducible interactive analysis and graphics of microbiome census data. PLoS One. 2013;8:e61217. 10.1371/JOURNAL.PONE.0061217 23630581 PMC3632530

[bib43] Morgan M, Anders S, Lawrence M et al. ShortRead: a bioconductor package for input, quality assessment and exploration of high-throughput sequence data. Bioinformatics. 2009;25:2607–8. 10.1093/BIOINFORMATICS/BTP450 19654119 PMC2752612

[bib44] Nahurira R, Ren L, Song J et al. Degradation of Di(2-Ethylhexyl) phthalate by a novel *Gordonia alkanivorans* strain YC-RL2. Curr Microbiol. 2017;74:309–19. 10.1007/S00284-016-1159-9/28078431

[bib45] Nshimiyimana JB, Khadka S, Zou P et al. Study on biodegradation kinetics of di-2-ethylhexyl phthalate by newly isolated halotolerant *Ochrobactrum anthropi* strain.L1 -W. BMC Res Notes. 2020;13:1–6. 10.1186/S13104-020-05096-0 PMC7247211 32448295

[bib46] Oberbeckmann S, Kreikemeyer B, Labrenz M. Environmental factors support the formation of specific bacterial assemblages on microplastics. Front Microbiol. 2018;8:308230. 10.3389/FMICB.2017.02709 PMC5785724 29403454

[bib47] Oberbeckmann S, Labrenz M. Marine microbial assemblages on microplastics: diversity, adaptation, and role in degradation. Annu Rev Mar Sci. 2020;12:209–32. 10.1146/ANNUREV-MARINE-010419-010633/1 31226027

[bib48] Paluselli A, Fauvelle V, Galgani F et al. Phthalate release from plastic fragments and degradation in seawater. Environ Sci Technol. 2018;53:166–75. 10.1021/ACS.EST.8B05083 30479129

[bib49] Prince RC, Butler JD, Redman AD. The rate of crude oil biodegradation in the sea. Environ Sci Technol. 2017;51:1278–84. 10.1021/ACS.EST.6B03207 27700058

[bib50] Qadeer A, Anis M, Warner GR et al. Global environmental and toxicological data of emerging plasticizers: current knowledge, regrettable substitution dilemma, green solution and future perspectives. Green Chem. 2024;26:5635–83. 10.1039/D3GC03428C 39553194 PMC11566117

[bib51] Quast C, Pruesse E, Yilmaz P et al. The SILVA ribosomal RNA gene database project: improved data processing and web-based tools. Nucleic Acids Res. 2013;41:D590–6. 10.1093/NAR/GKS1219 23193283 PMC3531112

[bib52] Rani M, Shim WJ, Han GM et al. Qualitative analysis of additives in plastic marine debris and its new products. Arch Environ Contam Toxicol. 2015;69:352–66. 10.1007/s00244-015-0224-x 26329499

[bib53] Ridley RS, Conrad RE, Lindner BG et al. Potential routes of plastics biotransformation involving novel plastizymes revealed by global multi-omic analysis of plastic associated microbes. Sci Rep. 2024;14:1–19. 10.1038/S41598-024-59279-X 38627476 PMC11021508

[bib54] Roager L, Sonnenschein EC. Bacterial candidates for colonization and degradation of marine plastic debris. Environ Sci Technol. 2019;53:11636–43. 10.1021/ACS.EST.9B02212/31557003

[bib55] Rowdhwal SSS, Chen J. Toxic effects of di-2-ethylhexyl phthalate: an overview. Biomed Res Int. 2018;2018:1750368. 10.1155/2018/1750368 29682520 PMC5842715

[bib56] Saeng-kla K, Mhuantong W, Termsaithong T et al. Biodegradation of di-2-ethylhexyl phthalate by mangrove sediment microbiome impacted by chronic plastic waste. Mar Biotechnol. 2025;27:1–12. 10.1007/S10126-024-10399-5/39625614

[bib57] Salinas J, Martínez-Gallardo MR, Jurado MM et al. Microbial consortia for multi-plastic waste biodegradation: selection and validation. Environ Technol Innov. 2024;36:103887. 10.1016/J.ETI.2024.103887

[bib58] Salvador M, Abdulmutalib U, Gonzalez J et al. Microbial genes for a circular and sustainable bio-PET economy. Genes. 2019;10:373. 10.3390/GENES10050373 31100963 PMC6562992

[bib59] Scales BS, Cable RN, Duhaime MB et al. Cross-hemisphere study reveals geographically ubiquitous, plastic-specific bacteria emerging from the rare and unexplored biosphere. mSphere. 2021;6:00851–20. 10.1128/MSPHERE.00851-20/PMC8265672 34106771

[bib60] Schaerer L, Putman L, Bigcraft I et al. Coexistence of specialist and generalist species within mixed plastic derivative-utilizing microbial communities. Microbiome. 2023;11:1–15. 10.1186/S40168-023-01645-4 37838714 PMC10576394

[bib61] Schaerer LG, Aloba S, Wood E et al. Enriched microbial consortia from natural environments reveal core groups of microbial taxa able to degrade terephthalate and terphthalamide. PLoS One. 2024;19:e0315432. 10.1371/JOURNAL.PONE.0315432 39729501 PMC11676569

[bib62] Sheridan EA, Fonvielle JA, Cottingham S et al. Plastic pollution fosters more microbial growth in lakes than natural organic matter. Nat Commun. 2022;13:1–9. 10.1038/S41467-022-31691-9 35882837 PMC9325981

[bib86_920_175926] Simon, M., Scheuner, C., Meier-Kolthoff, J. P., Brinkhoff, T., Wagner-Döbler, I., Ulbrich, M., Markus G. Phylogenomics of Rhodobacteraceae reveals evolutionary adaptation to marine and non-marine habitats. The ISME journal. 2017;11:1483–1499. 10.1038/ismej.2016.198 28106881 PMC5437341

[bib63] Singleton SL, Davis EW, Arnold HK et al. Identification of rare microbial colonizers of plastic materials incubated in a coral reef environment. Front Microbiol. 2023;14:1259014. 10.3389/FMICB.2023.1259014 37869676 PMC10585116

[bib64] Taudière A. MiscMetabar: an R package to facilitate visualization and reproducibility in metabarcoding analysis. J Open Source Softw. 2023;8:6038. 10.21105/JOSS.06038

[bib65] Temporiti MEE, Nicola L, Nielsen E et al. Fungal enzymes involved in plastics biodegradation. Microorganisms. 2022;10:1180. 10.3390/MICROORGANISMS10061180 35744698 PMC9230134

[bib66] Virtanen P, Gommers R, Oliphant TE et al. SciPy 1.0: fundamental algorithms for scientific computing in Python. Nat Methods. 2020;17:261–72. 10.1038/S41592-019-0686-2 32015543 PMC7056644

[bib67] Wickham H. ggplot2. Wiley Interdiscip Rev Comput Stat. 2011;3:180–5. 10.1002/WICS.147

[bib68] Wiesinger H, Wang Z, Hellweg S. Deep dive into plastic monomers, additives, and processing aids. Environ Sci Technol. 2021;55:9339–51. 10.1021/ACS.EST.1C00976 34154322

[bib69] Wilkes RA, Zhou N, Carroll AL et al. Mechanisms of polyethylene terephthalate pellet fragmentation into nanoplastics and assimilable carbons by Wastewater Comamonas. Environ Sci Technol. 2024;58:19338–52. 10.1021/ACS.EST.4C06645 39360733 PMC11526368

[bib70] Williams AT, Rangel-Buitrago N. The past, present, and future of plastic pollution. Mar Pollut Bull. 2022;176:113429. 10.1016/J.MARPOLBUL.2022.113429 35217417

[bib71] Wirnitzer U, Rickenbacher U, Katerkamp A et al. Systemic toxicity of di-2-ethylhexyl terephthalate (DEHT) in rodents following four weeks of intravenous exposure. Toxicol Lett. 2011;205:8–14. 10.1016/J.TOXLET.2011.04.020 21616130

[bib72] Wowkonowicz P. Phthalates in the environment: their toxicology and associated risk to humans. Ochrona Srodowiska i Zasobow Naturalnych. 2023;34:1–12. 10.2478/OSZN-2023-0001

[bib73] Wright RJ, Bosch R, Langille MGI et al. A multi-OMIC characterisation of biodegradation and microbial community succession within the PET plastisphere. Microbiome. 2021;9:1–22. 10.1186/S40168-021-01054-5 34154652 PMC8215760

[bib74] Xu J, Lu Q, de Toledo RA et al. Degradation of di-2-ethylhexyl phthalate (DEHP) by an indigenous isolate *Acinetobacter* sp. SN13. Int Biodeterior Biodegradation. 2017;117:205–14. 10.1016/J.IBIOD.2017.01.004

[bib75] Xu Y, Xiong B, Huang Y-MM et al. Exploring additives beyond phthalates: release from plastic mulching films, biodegradation and occurrence in agricultural soils. Sci Total Environ. 2024;918:170763. 10.1016/J.SCITOTENV.2024.170763 38336072

[bib76] Xu Z, Zhang J, Qi R et al. Complex release dynamics of microplastic additives: an interplay of additive degradation and microplastic aging. J Hazard Mater. 2025;490:137711. 10.1016/J.JHAZMAT.2025.137711 40024124

[bib77] Yew M, Yang Y, Wang Q et al. High-throughput screening strategies for plastic-depolymerizing enzymes. Trends Biotechnol. 2025;43:1550–65. 10.1016/J.TIBTECH.2024.12.008 39843328

[bib78] Yu T, Xiaodong L, Ai J et al. Microbial community succession during crude oil-degrading bacterial enrichment cultivation and construction of a degrading consortium. Front Microbiol. 2022;13:1044448. 10.3389/FMICB.2022.1044448/36406435 PMC9672818

[bib79] Zadjelovic V, Erni-Cassola G, Obrador-Viel T et al. A mechanistic understanding of polyethylene biodegradation by the marine bacterium Alcanivorax. J Hazard Mater. 2022;436:129278. 10.1016/J.JHAZMAT.2022.129278 35739790

[bib80] Zhan W, Yang H, Zhang J et al. Association between co-exposure to phenols and phthalates mixture and infertility risk in women. Environ Res. 2022;215:114244. 10.1016/J.ENVRES.2022.114244 36058272

[bib81] Zimmermann L, Bartosova Z, Braun K et al. Plastic products leach chemicals that induce in vitro toxicity under realistic use conditions. Environ Sci Technol. 2021;55:11814–23. 10.1021/ACS.EST.1C01103 34488348 PMC8427741

[bib82] Zolfaghari M, Drogui P, Seyhi B et al. Occurrence, fate and effects of Di (2-ethylhexyl) phthalate in wastewater treatment plants: a review. Environ Pollut. 2014;194:281–93. 10.1016/J.ENVPOL.2014.07.014 25091800

[bib83] Zumstein MT, Kohler HPE, McNeill K et al. High-throughput analysis of enzymatic hydrolysis of biodegradable polyesters by monitoring cohydrolysis of a polyester-embedded fluorogenic probe. Environ Sci Technol. 2017;51:4358–67. 10.1021/ACS.EST.6B06060 28140581

[bib84] Zurier HS, Goddard JM. A high-throughput expression and screening platform for applications-driven PETase engineering. Biotechnol Bioeng. 2023;120:1000–14. 10.1002/BIT.28319 36575047

