# Supplementary material for: Plastic additives enrich diverse bacterial communities which show the hallmarks of plastic degradation
Source: Sustain Microbiol. 2026 Jan 28;3(1):qvag003. doi: 10.1093/sumbio/qvag003 (PMC13375310; doi:10.1093/sumbio/qvag003)
Supplement: Supplementary file 1 [file qvag003_Supplemental_File.pdf]

Supplementary Information

To 'Plastic additives enrich diverse bacterial communities which show the hallmarks of plastic degradation'.

Supplementary Figures

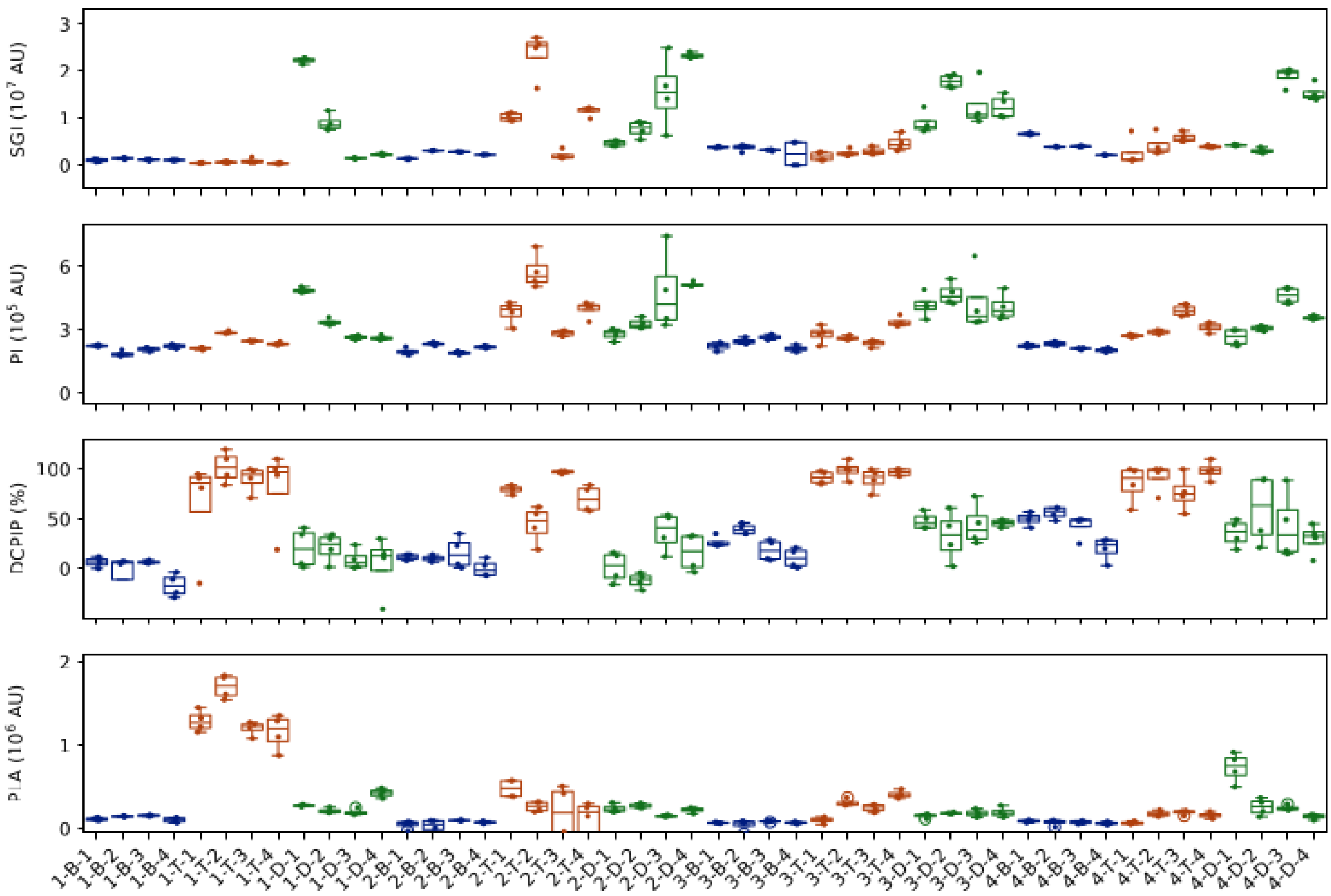

Supplementary Figure 1. Median assay signal at day 22. Data for each of four biological replicates, for each substrate-inocula condition, boxplots summarise technical replicate data (N=4). Data is coloured by substrate: negative control (B, blue), tetradecane (T, orange) and DEHT (D, green) and ordered by inocula: 1 (Chessel Bay), 2 (Swansea Bay), 3 (Water Treatment Works), 4 (Recycling Plant). The last number of the x axis labels indicates the replicate number.

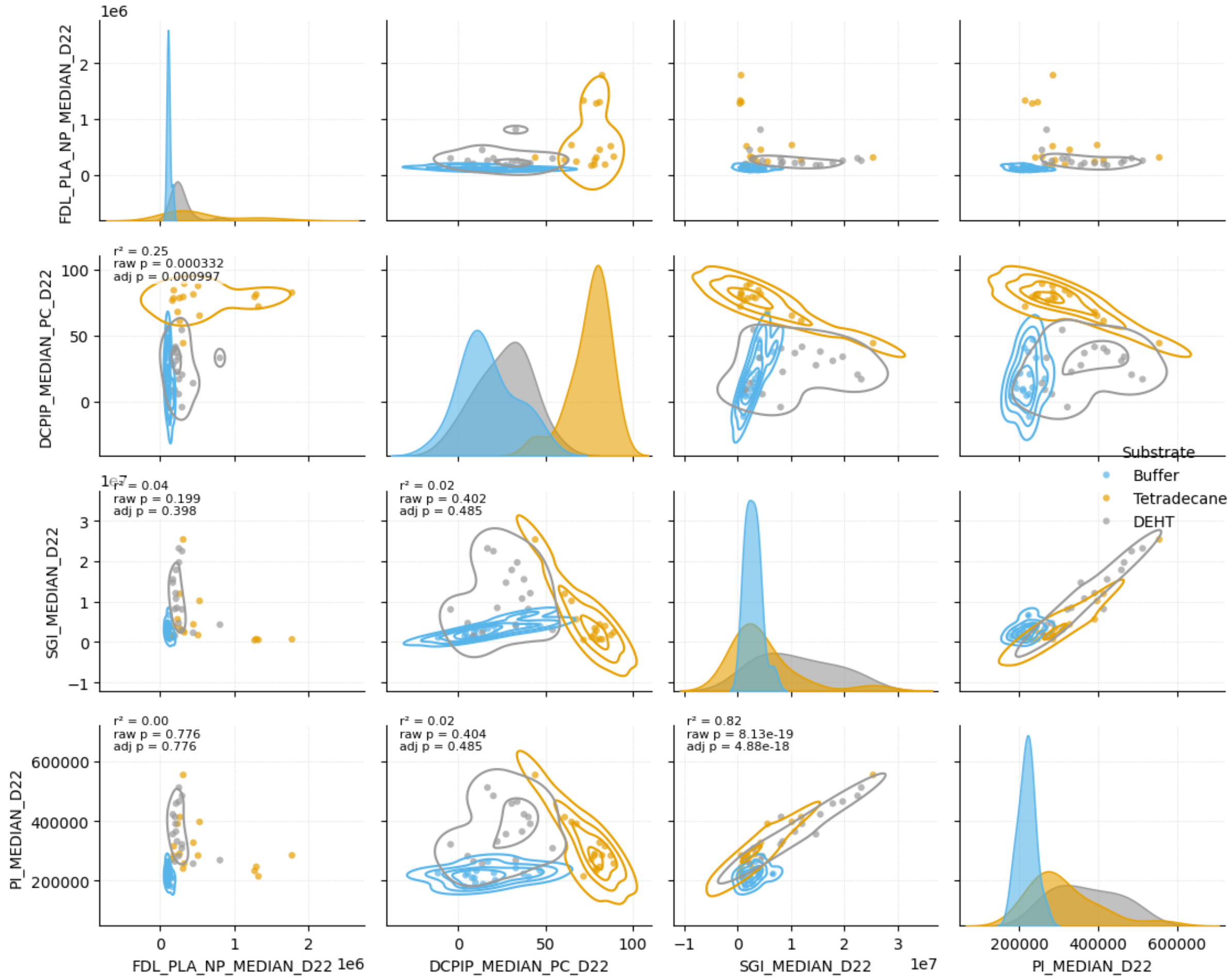

Supplementary Figure 2. Pairwise correlation matrix of assay endpoints at day 22. Scatterplots and kernel density estimates (KDEs) are shown for each pair of assay readouts (FDL, DCPIP, SGI, PI). Points are colored by substrate. Diagonal panels display univariate KDE distributions. Pearson correlation coefficients (r^2) and associated p-values (both raw and adjusted for multiple testing using the Benjamini–Hochberg false discovery rate) are annotated within the bottom off-diagonal panels for the entire datasets in each panel.

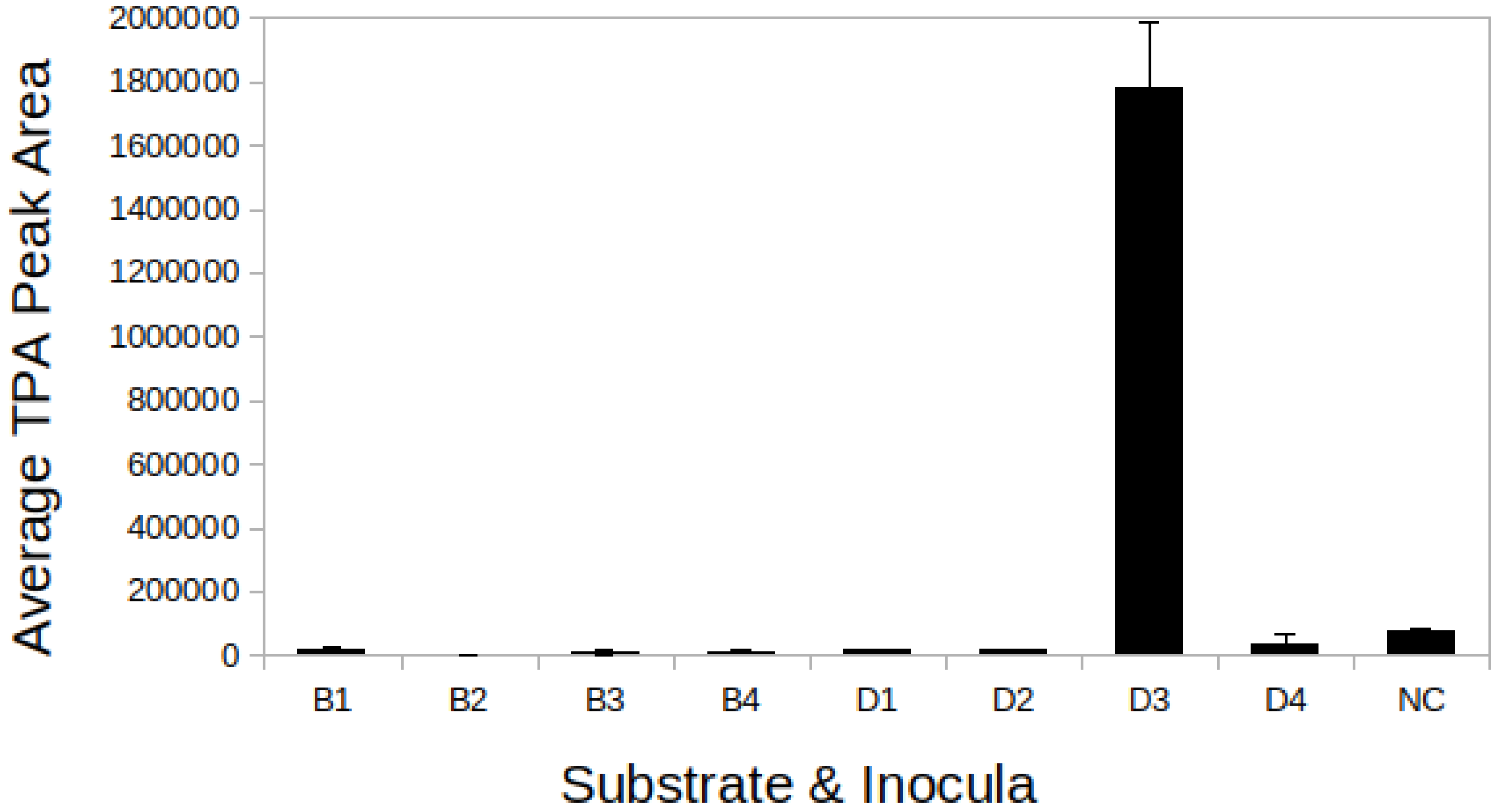

	MHET	NEG ION
Rt (mins)	CONC(ng)	AREA
9.75	100	168031
	250	425414
	500	842823
	1000	1444571

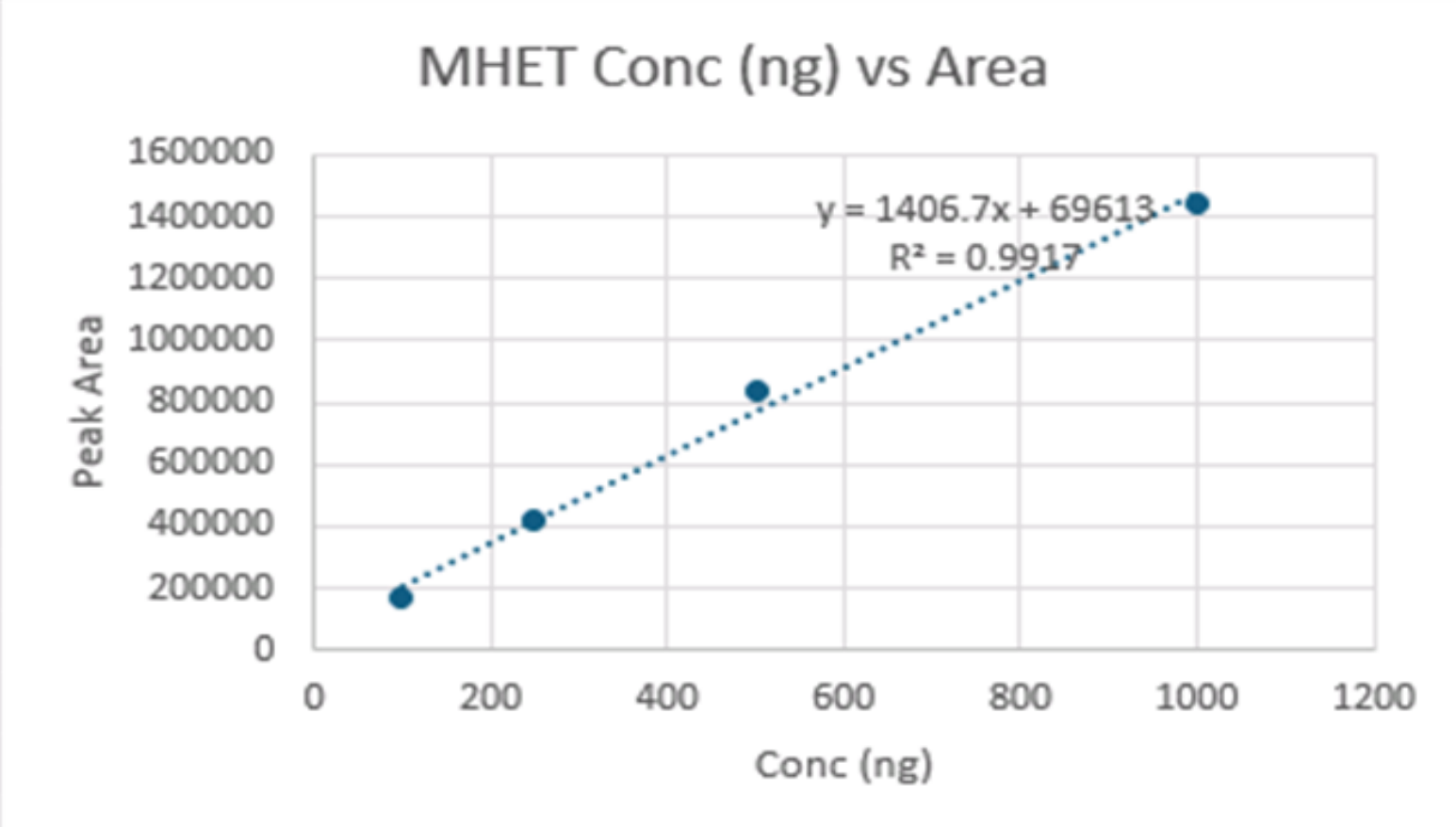

	TPA	NEG ION
Rt (mins)	CONC(ng)	AREA
9.34	500	268032
	1000	674521
	2500	888238

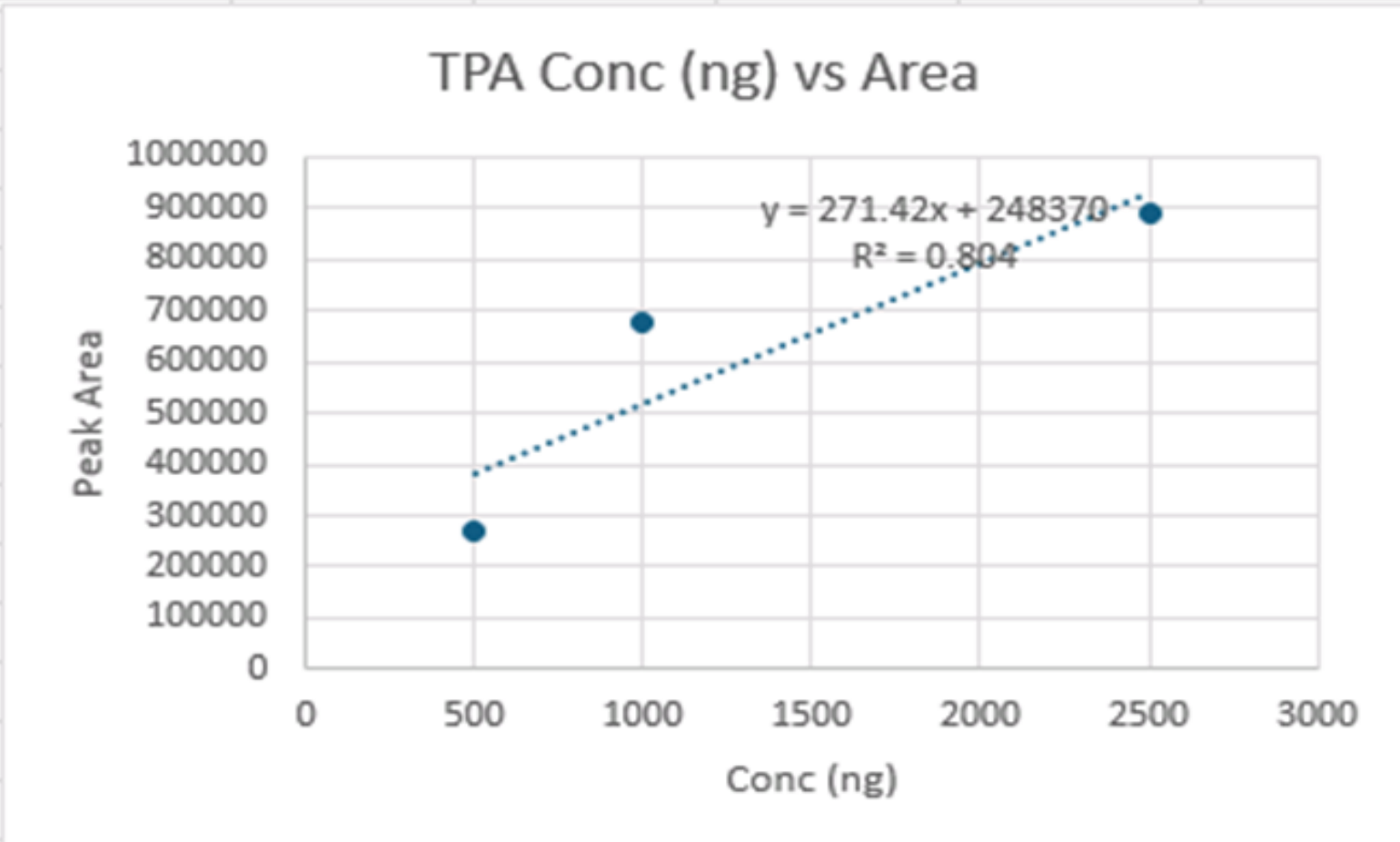

Supplementary Figure 3. A. Average terephthalic acid (TPA) peak area and standard deviation of three biological replicates (1-3) from the four inocula (1 (Chessel Bay), 2 (Swansea Bay), 3 (Wastewater Plant), 4 (Recycling Plant)) enriched on buffer (B) or DEHT (D), as well as a negative control (NC). B. Calibration curve indicating correlation between terephthalic acid concentration (ng) and peak area.

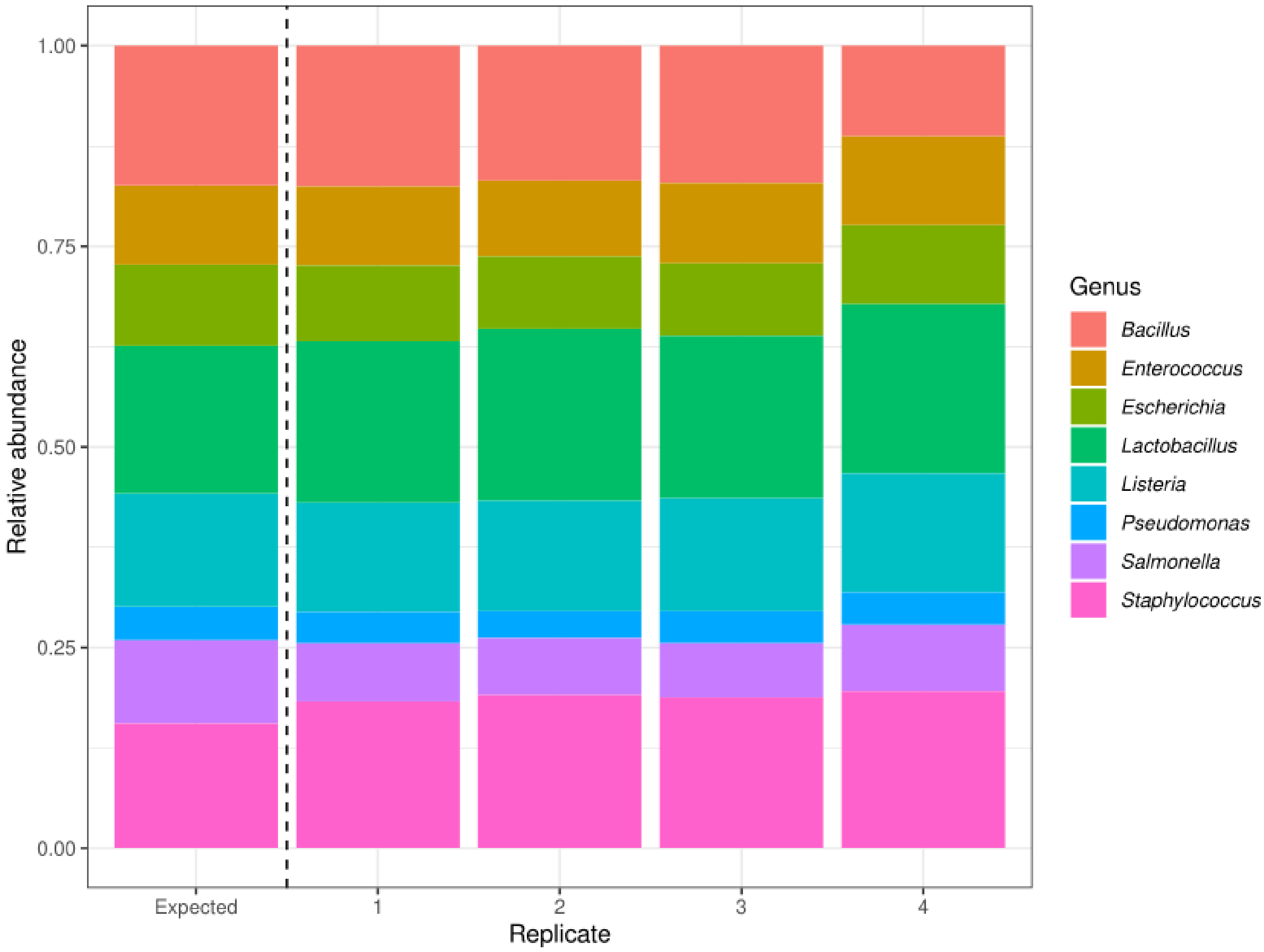

Supplementary Figure 4. Sequencing read distributions for positive control samples (N=4). The expected distribution of 16S rRNA for the Zymobiomics Microbial Community Standard (Zymo D6306) is shown and is as follows: *Pseudomonas aeruginosa* (4.2%), *Escherichia coli* (10.1%), *Salmonella enterica* (10.4%), *Lactobacillus fermentum* (18.4%), *Enterococcus faecalis* (9.9%), *Staphylococcus aureus* (15.5%), *Listeria monocytogenes*(14.1%) and *Bacillus subtilis*(17.4%).

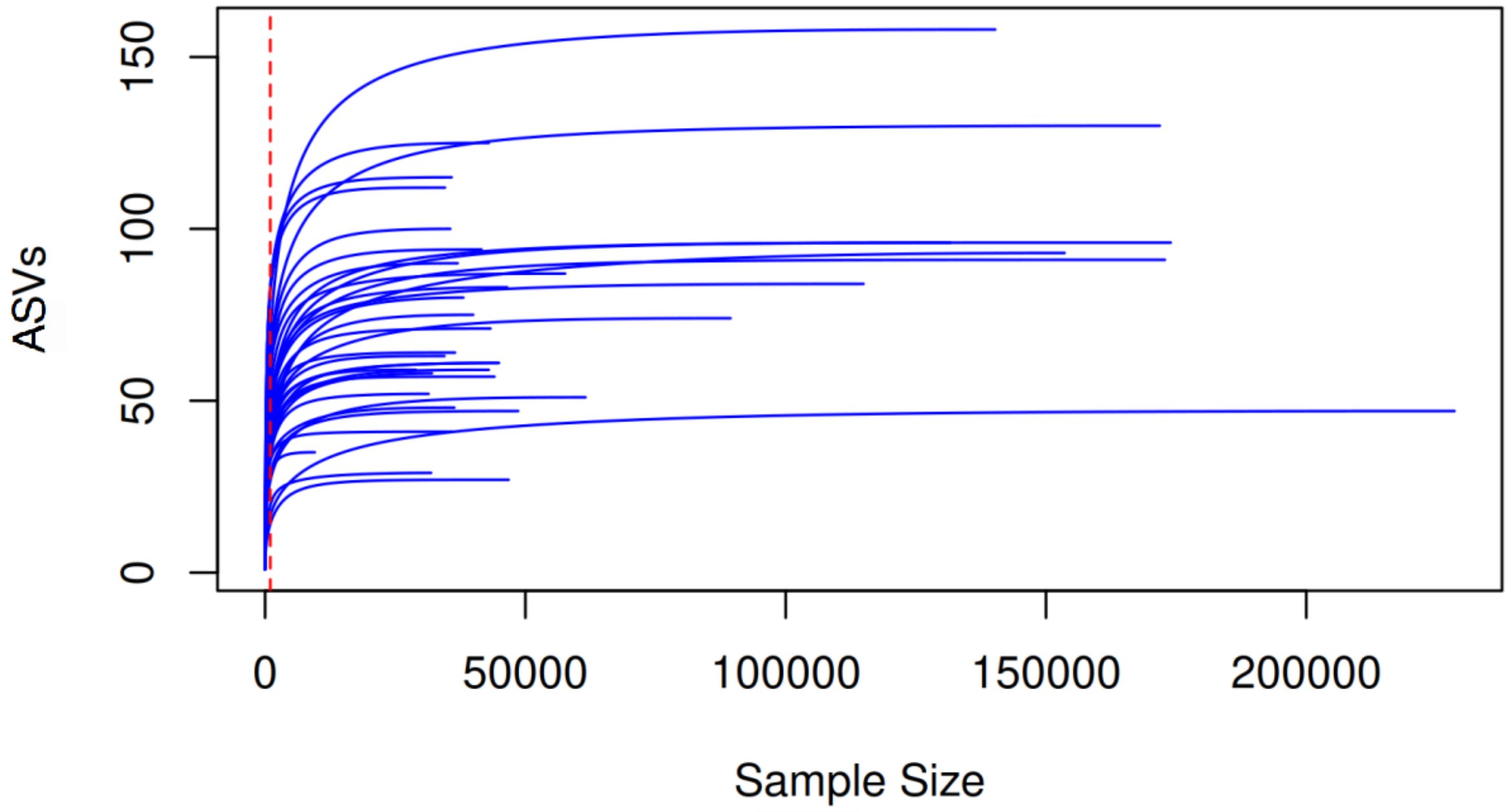

Supplementary Figure 5. Rarefaction curves as ASVs over sampling size (vertical dashed red line at x = 1000) of each biological replicate (36 samples in total).

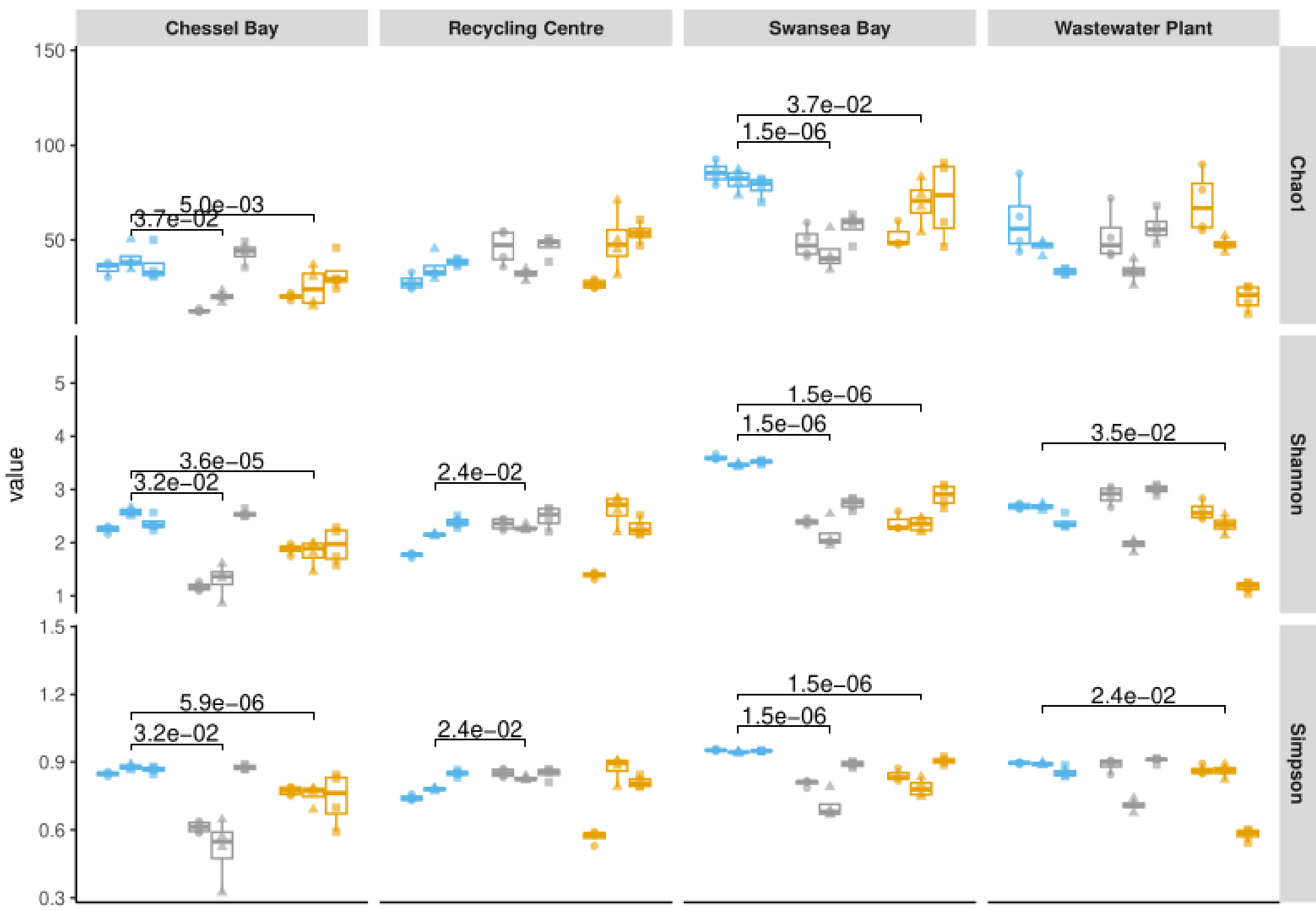

Supplementary Figure 6. Alpha diversity (Chao1, Shannon, Simpson, value specified on y-axis) of microbial communities, of three biological replicates grouped by the four environmental inocula (Chessel Bay, Swansea Bay, Wastewater Plant, Recycling Plant), coloured by substrate (blue = negative control, grey = DEHT, yellow = tetradecane).

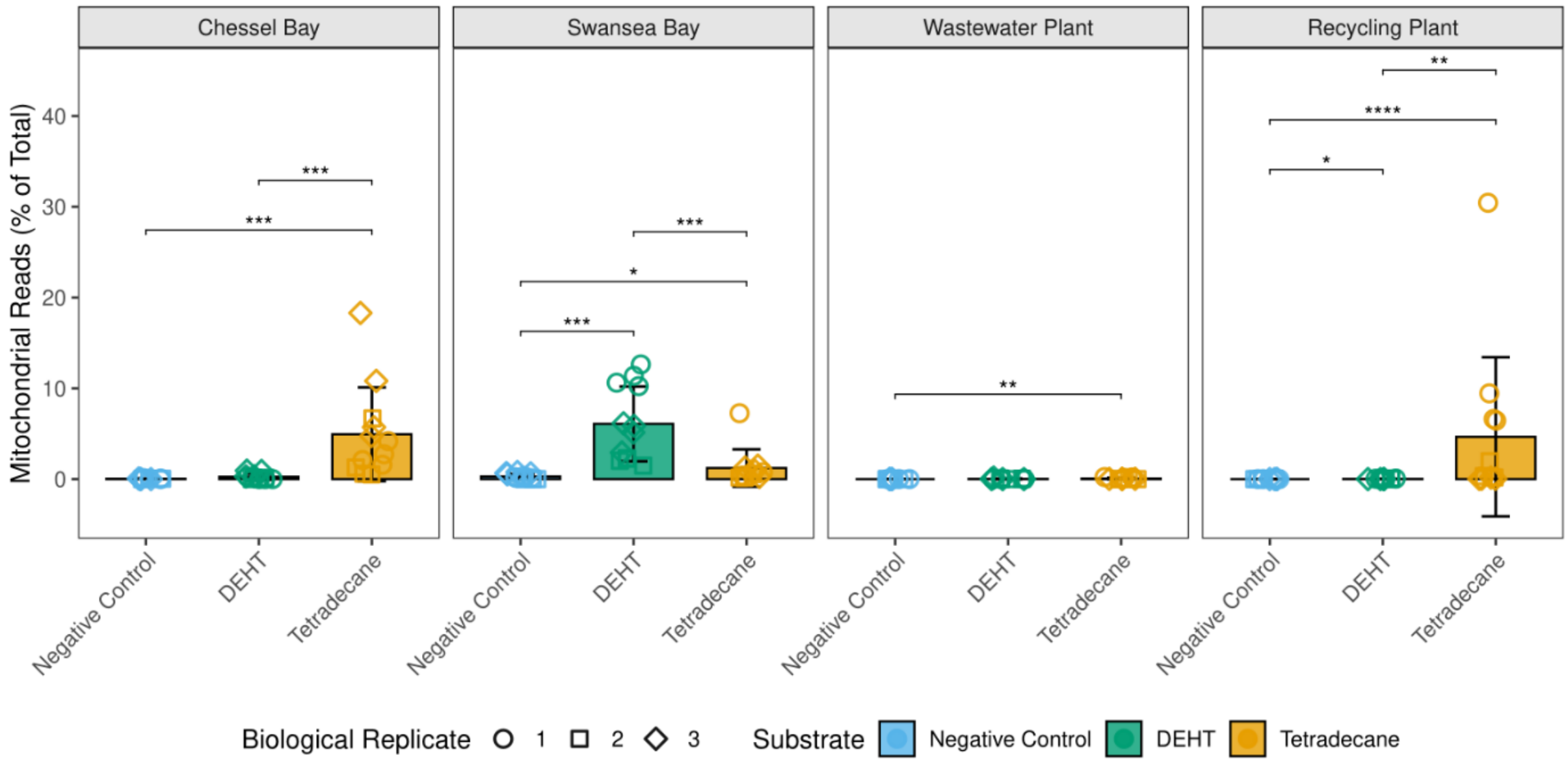

Supplementary Figure 7. Relative abundance of mitochondrial reads across inocula and substrates. Bars represent the mean ± standard deviation (SD) of mitochondrial reads (expressed as a percentage of total reads) for each substrate within each inoculum. Individual points show values for all biological and technical replicates, colored by substrate and shaped by biological replicate identity. Statistical significance between substrates within each inoculum was assessed using pairwise Wilcoxon rank-sum tests, with Benjamini–Hochberg–adjusted *p*-values indicated above bars ($p < 0.05$ = *, $p < 0.01$ = **, $p < 0.001$ = ***).

Supplementary Tables

Supplementary Table 1. Temporal trends in assay responses within each substrate treatment. Summary of temporal trends in microbial assay responses between sequential sampling points (Day 7 vs Day 2 and Day 22 vs Day 7) for each substrate and assay type (SYBR Green I, PI, FDL, DCPIP). For each substrate, the table reports the median ± standard deviation (SD) at each time point, fold change (FC) between time points, unadjusted and Benjamini–Hochberg adjusted p-values (padj), and direction of change (↑ increase, ↓ decrease, or no change). Pairwise comparisons were performed using two-sided Mann–Whitney U tests. Statistical significance is denoted as padj ≤ 0.05 (*), ≤ 0.01 (**), ≤ 0.001 (***). These analyses identify time-dependent shifts in microbial biomass, membrane integrity, esterase activity, and redox activity under different substrate conditions.

Supplementary Table 2. Comparisons of each substrate versus the negative control at each time point. Pairwise comparisons of median assay responses for DEHT and tetradecane treatments relative to the corresponding negative-control samples at each time point. For each assay, the table lists the substrate median ± SD, fold-change (median treatment / median control), Mann–Whitney U test p-values, Benjamini–Hochberg adjusted p-values, and direction of effect (↑ increase, ↓ decrease, or no change). Significance thresholds are padj ≤ 0.05 (*), ≤ 0.01 (**), ≤ 0.001 (***). These data indicate substrate-specific stimulation or inhibition of microbial activity relative to unamended controls.

Supplementary Table 3. Inoculum-specific comparisons versus the negative control. Inoculum-specific comparisons of assay responses for each substrate treatment versus the corresponding negative-control samples at each time point. For each inoculum, the table provides median assay values, fold-change relative to controls, raw and adjusted p-values (Benjamini–Hochberg correction), and direction of response (↑ increase, ↓ decrease, – no change). Pairwise comparisons were conducted using two-sided Mann–Whitney U tests on biological replicate-level data rather than medians to preserve statistical power. Significance thresholds are padj ≤ 0.05 (*), ≤ 0.01 (**), ≤ 0.001 (***). This table highlights variability among environmental inocula in their responses to DEHT and tetradecane exposure.

Supplementary Table 4. Assay measurements (median across technical replicates) for cell biomass (SYBR Green I fluorescence), dead cells (propidium iodide (PI) fluorescence), esterase activity (fluorescein diacetate (FDL) fluorescence) and redox activity (DCPIP reduction) for each Inoculum × Substrate × Time × Biological Replicate combination. This data was used to generate Supplementary Table 1-3 and Figure 1.

Supplementary Table 5. Number of sequencing reads for technical replicates and assay measurements for cell biomass (SYBR Green I fluorescence), dead cells (propidium iodide (PI) fluorescence), esterase activity (fluorescein diacetate (FDL) fluorescence) and redox activity (DCPIP reduction) for each Inoculum × Substrate × Time × Biological Replicate x Technical Replicate combination. This data was used to generate Supplementary Table 4.

Supplementary Methods

Preparation of Tris-minimal media

First, stock solutions were prepared. Solution A was prepared by combining ammonium chloride (20 g, Fisher Scientific 11314609) with magnesium sulfate heptahydrate (5 g, Fisher Scientific 11411828) and calcium chloride dihydrate (2.5 g, Fisher Scientific 10786634) in milliQ water (500 mL). Phosphate Buffer II was prepared by combining dipotassium hydrogen orthophosphate (10.8 g, Fisher Scientific 16584204) with monopotassium phosphate (5.6 g, Fisher Scientific 10757214) in milliQ water (100 mL). Stock solutions were stored in the fridge and tris-minimal media was prepared (Gorman and Levine, 1965) by combining 1M Tris base (20 mL) with Phosphate Buffer II (1.0 mL), Solution A (10 mL), trace Metal Mix A5 with Co (1.0 mL, SLS 92949-25ML). This solution was made up to a total volume of 1000 mL with milliQ water following adjustment of pH to 7.0 using hydrochloric acid. Once made, the buffer was autoclaved.

Mass Spectrometry

See attached document for full protocol, chromatograms and spectra.
